# Cyanotoxin Occurrence and Diversity in 98 Cyanobacterial Blooms from Swedish Lakes and the Baltic Sea

**DOI:** 10.3390/md22050199

**Published:** 2024-04-27

**Authors:** Caroline Dirks, Paolo Cappelli, Maria Blomqvist, Susanne Ekroth, Malin Johansson, Max Persson, Stina Drakare, Heidi Pekar, Aida Zuberovic Muratovic

**Affiliations:** 1 Swedish Food Agency, P.O. Box 622, SE-751 26 Uppsala, Sweden; 2 Wageningen Food Safety Research, P.O. Box 230, 6700AE Wageningen, The Netherlands; 3 Department of Aquatic Sciences and Assessment, Swedish University of Agricultural Sciences, P.O. Box 7050, SE-750 07 Uppsala, Sweden; 4 Stockholm Vatten och Avfall, Bryggerivägen 10, SE-106 36 Stockholm, Sweden

**Keywords:** cyanotoxins, cyanobacteria, blooms, analysis, survey, LC-MS/MS

## Abstract

The Drinking Water Directive (EU) 2020/2184 includes the parameter microcystin LR, a cyanotoxin, which drinking water producers need to analyze if the water source has potential for cyanobacterial blooms. In light of the increasing occurrences of cyanobacterial blooms worldwide and given that more than 50 percent of the drinking water in Sweden is produced from surface water, both fresh and brackish, the need for improved knowledge about cyanotoxin occurrence and cyanobacterial diversity has increased. In this study, a total of 98 cyanobacterial blooms were sampled in 2016–2017 and identified based on their toxin production and taxonomical compositions. The surface water samples from freshwater lakes throughout Sweden including brackish water from eight east coast locations along the Baltic Sea were analyzed for their toxin content with LC-MS/MS and taxonomic composition with 16S rRNA amplicon sequencing. Both the extracellular and the total toxin content were analyzed. Microcystin’s prevalence was highest with presence in 82% of blooms, of which as a free toxin in 39% of blooms. Saxitoxins were found in 36% of blooms in which the congener decarbamoylsaxitoxin (dcSTX) was detected for the first time in Swedish surface waters at four sampling sites. Anatoxins were most rarely detected, followed by cylindrospermopsin, which were found in 6% and 10% of samples, respectively. As expected, nodularin was detected in samples collected from the Baltic Sea only. The cyanobacterial operational taxonomic units (OTUs) with the highest abundance and prevalence could be annotated to *Aphanizomenon* NIES-81 and the second most profuse cyanobacterial taxon to *Microcystis* PCC 7914. In addition, two correlations were found, one between *Aphanizomenon* NIES-81 and saxitoxins and another between *Microcystis* PCC 7914 and microcystins. This study is of value to drinking water management and scientists involved in recognizing and controlling toxic cyanobacteria blooms.

## 1. Introduction

Cyanobacteria prosper in many water resources worldwide, both in fresh and brackish water systems, where they represent a nuisance and a threat to public health, including in Swedish lakes and the Baltic Sea [1,2]. Research on cyanobacterial blooms (cyanoblooms) from recent years also shows that they occur earlier and have become more extensive than 40 years ago [3]. The increasing frequency and intensity of cyanoblooms in lakes, rivers, and seas have been linked to input of nutrients, like nitrogen and phosphorus (eutrophication) due to urban, industrial, and agricultural activities, in combination with elevated average water temperatures (global warming) [4]. The proliferation of cyanobacteria during a bloom formation increases the cyanobacterial biomass over a relatively short period of time (days to weeks) and is usually dominated by one or a few cyanobacteria taxa of the phytoplankton community [5,6]. This causes major problems by straining the water treatment plants, but also for the recreational and tourism activities by impelling bathing places to close.

In a phytoplankton community, toxic and non-toxic cyanobacteria co-exist, although during a cyanobloom toxic taxa might proliferate producing potent cyanotoxins that are released to the surrounding water after cell death [7]. As cyanoblooms may occur in different fresh water sources, there is a range of unintentional exposure routes where both humans and animals can be affected if the proliferating species produce cyanotoxins. Since cyanobacteria might produce various potent toxins [8], severe cyanotoxin outbreaks represent a versatile problem for humans, domestic animals, and ecosystems where wild animals can suffer from serious diseases or even die after ingesting water that contains toxin-producing cyanobacteria [9,10,11,12,13]. For humans, the illness associated with the exposure to cyanotoxins is manifested by headaches and nausea with vomiting and diarrhea [14]. Some of cyanotoxins cause liver damage and are tumor-promoters [15,16] while others are neurotoxins that have a paralytic effect [17,18,19]. A toxin-producing cyanobloom becomes thus an extensive societal issue when it hits the surface water reservoirs that are used for the production of drinking water. Due to the underlined toxicity, cyanotoxins of prime importance to monitor are microcystins (MCs), nodularin (NOD), cylindrospermopsin (CYN), anatoxins (ATXs, anatoxin-a and homoanatoxin-a, hATX), and saxitoxins (STXs). As the knowledge about the prevalence of cyanotoxins and the diversity of the producing species in cyanoblooms in Sweden is still limited, the aim of this study was to identify their occurrence and the toxin profiles to improve the insight into the bloom characteristics.

There are several types of methods that can be applied to evaluate cyanoblooms. Traditionally, light microscopy is often used to assess cyanobacterial composition but not to identify toxic taxa [20,21]. More recently, 16S rRNA amplicon sequencing is used for the same purpose to determine the taxonomic composition of cyanoblooms [22,23,24]. The second most commonly applied approach is the biochemical assay, Enzyme-Linked Immunosorbent Assay (ELISA) [20,25,26], to screen for cyanotoxin presence in water samples, a method that possesses a higher sensitivity but is known for lower specificity in comparison to chemical methods [27]. Molecular methods based on polymerase chain reaction (PCR or quantitative PCR, qPCR) represent an alternative technique in the investigation of cyanoblooms to confirm the presence of genes encoding specific toxins. However, the limitation in using PCR methods for this purpose is that the confirmed presence of a toxin gene does not guarantee that the gene actually has been expressed, i.e., that the cyanobacterium has produced the toxin [28].

In general, mass spectrometry (MS)-based methods have become more commonly applied in the analysis of cyanotoxins in recent years compared to other techniques [29,30,31,32,33], where Ultra-Performance Liquid Chromatography (UPLC-MS/MS) has been most often preferred for detection with the quantification of cyanotoxins in various sample matrices [34,35,36,37].

In the present study, cyanoblooms that occurred in Sweden during the summer seasons of 2016 and 2017 are studied. Samples from ninety-eight cyanoblooms were collected from a wide spatial range representing different types of aquatic environments (lakes, bays, rivers, swamps, harbors, and bathing places), including four sources of drinking water. The sampling survey was a community-driven effort that involved advertising through various media such as conferences, advertisements, and through Facebook’s advanced location targeting.

The aim of the study was to selectively and quantitatively analyze the most interesting cyanotoxin congeners in the collected samples using UPLC-MS/MS and Ultra-Performance Hydrophilic Interaction Liquid Chromatography with tandem mass spectrometry (UP-HILIC-MS/MS) [2,38]. In addition, the taxonomic compositions of cyanobacteria were studied with 16S rRNA amplicon sequencing, to determine the taxonomic composition of the bloom, including the cyanobacterial taxa [39]. Microscopy was also used for species identification, mostly to facilitate the initial assessments carried out by staff in drinking water production [40]. Using the combined method approaches, a temporal pattern in profiles of cyanotoxins and species could be revealed. Toxin groups and cyanobacteria from previously unmonitored surface water areas were successfully studied. The obtained data will help to promote safe drinking water production as well as to elucidate the geographical distribution of the cyanobacterial blooms in Sweden. Finally, the obtained results will also be of benefit to future studies of cyanoblooms and their toxins in Swedish surface waters towards the tailoring of efficient mitigation and preventive measures and handling strategies.

## 2. Results and Discussion

About half of the drinking water in Sweden is produced from lakes and rivers. Blooms of cyanobacteria in lakes are a recurring feature during the summer and occur throughout Sweden, from south to north, and can last from a few hours to several weeks [41]. In some lakes, toxic blooms of cyanobacteria are more frequent than in others. Toxic blooms can also occur in infiltration ponds from which surface water is infiltrated (purified through the ground). Before raw water becomes drinking water, it is purified in the water treatment plants with various treatment steps that help to reduce and remove particles and organic material. The present study was planned as the need has been recognized to increase the knowledge about the cyanobacteria diversity and cyanotoxin profiles in different surface waters throughout Sweden. The results would serve to create a basis for bringing forth recommendations in order to handle risks related to cyanotoxins in drinking water production.

### 2.1. Study Site Selection and Sampling Strategy

As the sampling strategy was composed during this study, it is considered to belong to the Results Section of this article (Supplementary Material S1).

**Figure 1:**
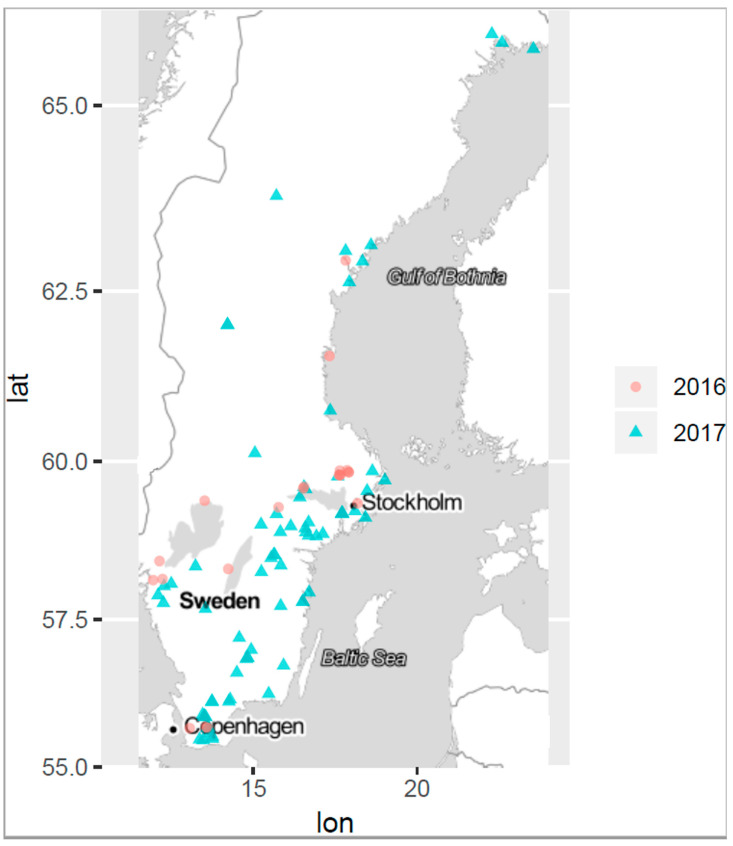
Map of sampling sites where the blooms were observed, 2016 (red dots) and 2017 (blue triangles). The most northern sampling site was at 65.9150371 N, 22.273922 E and the most southern sampling site at 55.4675428 N, 13.4682083 E (distance: 1255 km).

**Figure 2:**
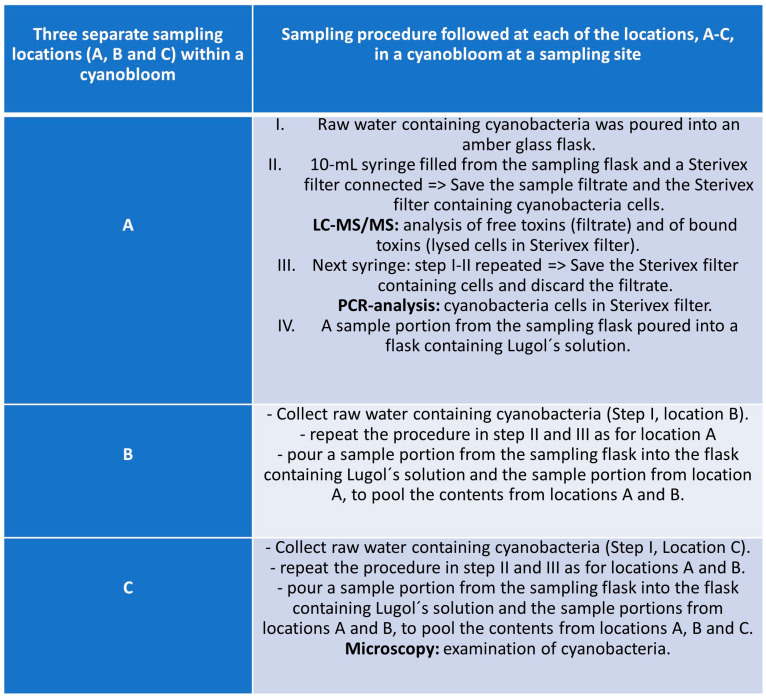
Flowchart of the sampling procedure (three replicate samplings), specifically developed for this study, used at each sampling site.

Often studies on cyanobacterial blooms collect samples from a single site assuming an even distribution of cyanobacterial species and cyanotoxins across the bloom. However, during a cyanobloom the cell densities of cyanobacteria are highly dependent on the current drift and the wind, which often cause high spatial variability across the water surface [42,43,44]. Within short time periods, cell and toxin concentrations at a site can change dramatically. Furthermore, toxin concentrations within a cyanobloom are influenced by a complex interplay of thermal decomposition, photolysis via UV radiation, and microbial degradation, which all can vary between different locations within the same water body [45,46]. Consequently, a single sample cannot be representative of the entire bloom and, therefore, cannot provide accurate information on the cyanobloom characteristics. Hence, multiple sampling within a cyanobloom is required for better understanding and ensuring robust results; thus, three separate samples were taken in each bloom in this study. The sites are depicted in the site map in Figure 1. The sampling was dictated by ongoing blooms regardless of the geographical location or time point during the summer seasons, June–October 2016 and 2017. For this study, a specific sampling protocol was developed which is briefly described in the flowchart of Figure 2, while the entire sampling procedure instruction is presented in Supplementary Material S1, and the microscopy examination followed the sampling, as a pre-analytical support to LC-MS/MS analysis, is presented in Supplementary Material S2.

### 2.2. Diversity of Cyanotoxin Congeners

Cyanotoxins were detected in ninety-eight out of more than one hundred samples collected in the study. A detailed overview of the results for each sample and sampling site is presented in Table S2 in Supplementary Material S2, which the sample names will be referring to throughout Section 2.2 and Section 2.3. Microcystin RR (MC-RR) was, by far, the most often detected congener, in 70% of samples, and in 19% of samples, it was present only as a free toxin, as shown in Table 1. The standard deviation calculated for the total found levels of MC-RR testifies that this toxin occurred within a wide concentration range. The next most frequently occurring toxin was microcystin LR (MC-LR) that was found in 55% of samples and in 10% of samples as a free toxin. MC-LR also stands out as the only congener detected in high concentrations as a free toxin (average: 148.5 µg/L). Microcystin RR [Dha7] (MC-RR [Dha7]) is the congener with the largest share occurring as a free toxin (in 20% of samples as a free toxin compared to 46% of the total share of samples in which it was detected). Other microcystins such as MC-YR and MC-RR [D-Asp3] + MC-RR [D-Asp3, (E)-Dhb7] seem to be represented in the samples to almost equal extents (in 36% and 39% of samples, respectively), although MC-YR is detected as a single and MC-RR [D-Asp3] + MC-RR [D-Asp3, (E)-Dhb7] as a double congener, since the method lacks specific m/z transitions for each of the two congeners in the pair. The remaining eleven microcystins detected are as follows: MC-LR [DAsp3] (22%), MC-WR (19%), MC (N-methyl-L)R (17%), MC-HilR (16%), MC-HtyR (13%), MC-LY (11%), MC-HphR [D-Asp3, (E)-Dhb7] (10%), MC-HtyR [D-Asp3,(E)-Dhb7] (9%), MC-LW and MC-LF with a share of 8% each, and MC-LA with the lowest frequency of occurrence and was detected in 3% of samples. All of the MC congeners were also detected as free toxins to different extents. The data on MCs presented in Table 1 reflects their dominance as the most common and diverse group of cyanotoxins in Swedish freshwaters of this study, represented in 82% of samples in which they were present either as single or multiple congeners together.

The frequency of occurrence was relatively low among the hydrophilic cyanotoxins such as anatoxin-a (ATX), homoanatoxin-a (h-ATX), and micropeptin 1106 as they were present in 1–6% of samples, in free forms or in total levels. The relevance of including the detection of micropeptin 1106 in this study, as a recent member of the group of cyanotoxins, and the toxicity of this substance were reported earlier [20]. The highly polar cylindrospermopsin (CYN) was present in 10% of samples, and in a free form in as many as 9%. These data are in coherence with what can be expected for CYN as a very stable toxin persistent in aquatic environments that, unlike other cyanotoxins, is mainly present as a free toxin (extracellular, up to 90%) [47,48]. CYN occurred in samples with toxins from one or more of the other cyanotoxin groups (MCs, STXs, ATXs), but never together with nodularin (NOD), in this study. NOD was present in 13% of samples, most of which were collected along the coast of the Baltic Sea or in bays connected to the Baltic Sea, where it is known to be a typical brackish water toxin produced by, amongst others, *Nodularia spumigena*. Among toxins in the saxitoxin group, the main congener, saxitoxin (STX), dominates with its presence in 45% of samples and occurs as a free toxin in a proportion of 36%. Consequently, STX is the second most abundant toxin in Swedish surface water samples and hence often detected in the same samples as MCs, although there are samples where STX is found as the only toxin (sites: Hå1, 17–08, 17–13, 17–18, 17–42, and 17–73; Table S2) or together with ATX, or ATX and NOD without any MCs (sites: 17–11 and 17–75; Table S2). Furthermore, within this study, decarbamoylsaxitoxin (dcSTX) was detected for the first time in Swedish surface water samples at four sampling sites (17–16, 17–22, 17–33, and 17–37), two of which are among the northernmost and the southernmost sampling sites of the study (Granön, Båtskärsnäs in the Baltic Sea and Ellestadssjön, Sjöbo). In addition to MCs, dcSTX was present together with STX as the dominating toxin analogue in all four samples, with an additional presence of CYN in one of these samples (site 17–37). The presence of hATX was modest in five samples (four sampling sites), mostly in single replicates of three samplings where it co-occurred with ATX-a.

### 2.3. Cyanotoxin Quantities

Toxin release from cyanobacteria increases during the terminal phase of the cyanobacterial growth and during the stationary phase of the cyanobloom. When a cyanobloom collapses meaning that the cyanobacteria die, an extensive toxin release can occur [47,49]. Hence, the concentration of the toxins found in a sample, including the variations in the free and the bound toxin levels, depends on the time point at which the sample was taken during the mass development of cyanobacteria. Consequently, for a study with non-continuous sampling at a certain sampling site over a longer period of time, the toxin concentration data found are temporary. At some of the sampling sites included in this study, in which the cyanoblooms are frequent and recurring annually, sampling was carried out more than once (e.g., at sites 17–57, 17–68, 17–60, and 17–62), and the toxin quantity data are presented per sampling date. In addition, sampling was carried out at more than one hundred different sites, although it could not always be approved in accordance with the sampling protocol, resulting in samples being inadequate for analysis and excluded from the study. The cyanotoxin quantities presented in Table S2 (Supplementary Material S2) are average values of the toxin concentrations found at three sampling locations in the same cyanobloom (the sampling site). For some of the sampling sites where the toxin quantification was achieved above the limit of quantification, LOQ (0.1 µg/L), in less than three locations within the same cyanobloom, the average toxin concentration was calculated applying “0” for the locations in which the toxin was below the LOQ or not detected. In this way, the exclusion of single positive sample replicates that are still relevant to visualize in the study was avoided to obtain more representative toxin concentration data. Consequently, some of the toxin average values shown in Table S2 are lower than the LOQ of the method. In the cases where toxins were detected below the LOQ for all three sampling locations, they are presented as <LOQ for the entire cyanobloom. Samples in which the toxins were detected beyond the calibration range of the method were diluted and reanalyzed. The highest concentrations measured in this study for the microcystin group peak at 26 mg/L (Så1) and decrease for sites 17–23, VSB1, 17–60, and 17–17. Other total concentrations found for MCs range between 0.1 and 222.4 µg/L and between 0.1 and 1382.1 µg/L as free toxins. Saxitoxins, mostly represented by the main analogue, STX, as described in Section 2.2, were generally present in concentrations below 25 µg/L, although higher concentrations were measured at three sites, i.e., 62.7 and 424.4 µg/L (sites: 17–21 and 17–22), including the highest concentration found for STX, 1891.0 µg/L (site 17–33). CYN was detected in concentrations up to 9.5 µg/L (site 17–37), while ATXs were present up to 4.2 µg/L (site Så1), of which hATX was detected in five samples and always at levels ≤LOQ of the method (up to 0.1 µg/L, calculated as the average of three samplings within the same sampling site).

The study shows a wide diversity among cyanotoxin analogues represented in Swedish surface waters, which makes the cyanotoxin profile complex even though it is differentiated between the studied sampling sites. The study also shows that the free toxin levels are generally much lower than the bound levels, being an important aspect of the drinking water production as the cyanobacterial cells are removed before the water enters the water treatment plants. However, the few quantitative data described above for MCs and STXs show that the free-toxin levels can also pose a huge challenge in drinking water production. Even though CYN and ATXs have been detected in lower concentrations, their water solubility and stability together with the high toxicity potential make them equally important to monitor in drinking water production as the other cyanotoxins found in this study.

### 2.4. Molecular Analysis

#### 2.4.1. Total Community Composition of the Samples

Based on 16S rRNA amplicon analysis, a total of 19.665 unique OTUs were identified in these fresh and brackish water cyanoblooms (Supplementary Material S3). However, only 516 OTUs had a relative abundance that was higher than 1% in at least one of the samples. The most ubiquitous taxon (OTU_12), present in 91% of the samples, was an *Alphaproteobacteria* of the family *Sphingomonadaceae*, i.e., not cyanobacteria. However, in only 40% of the sampling sites, its relative abundance was higher than 1%. In a sample from Lake Anten (site 17–21, replicate 3), the relative abundance of this taxon was as high as 80%. However, in another sample from the same site, its relative abundance was only 8%. The most species-rich sample was taken from Lake Vomb (site VS1), in which 4981 unique OTUs were detected. Heterotrophic bacteria are expected to benefit from the organic carbon produced by cyanobacteria during blooms.

#### 2.4.2. Cyanobacterial Community Composition

The cyanobacterial taxon OTU_10, annotated as *Aphanizomenon* NIES 81, was present in 88% of all freshwater blooms, making this the most ubiquitous cyanobacterial taxon across all sampled cyanoblooms, followed by *Cyanobium* PCC 6307 (OTU_13563) and *Microcystis* PCC 7914 (OTU_1), Table 2. The bloom with the most cyanobacterial species was Hornsundssjön (site 17–49) with an average of 212 unique cyanobacterial OTUs. The most abundant species in Hornsundssjön was *Aphanizomenon* NIES 81 (OTU_10) with a relative abundance of 17%. Seven cyanobacterial species in this bloom had an abundance greater than 1%. Overall, the community composition differed between the three samples taken from different locations within the same cyanobloom. One cyanobacterial community contained 10 different cyanobacterial OTUs of which all had a relative abundance higher than 1%. However, more often one or two cyanobacteria species would dominate the bloom. For instance, in Lake Vomb, more than 70% of the bloom consisted of Microcystis PCC 7914 (OTU_1), whereas the 61 other cyanobacterial taxa were present below 1% relative abundance (combined relative abundance: 3%). A study by Jankowiak et al. [50] also showed that *Microcystis* PCC 7914 was the dominating species in all the samples in their study carried out in two North American lakes.

**Figure 3:**
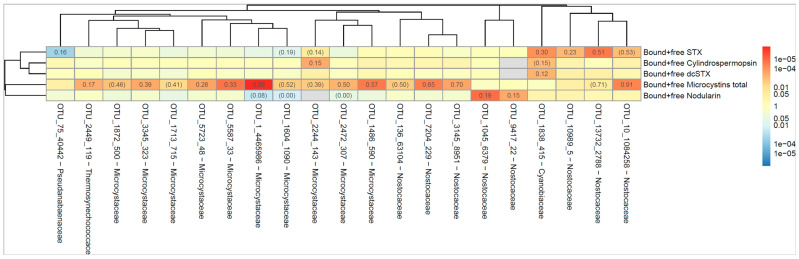
The heatmap shows the correlation between the occurrence of cyanobacteria (indicated as operational taxonomic unit, OTU) and the cyanotoxin. The numbers indicate the proportion of all samples where both the OTU and the toxin could be detected. Red color indicates that the correlation between OTU and toxin is positive and blue color if the correlation is negative. The darker the color, the lower the *p*-value (stronger association between OTU and toxin). The *p*-value was calculated according to Spearman’s correlation test. The dendrogram was generated using complete linkage hierarchical clustering based on a correlation distance.

The dendrogram in Figure 3 shows the correlation between the occurrence of cyanobacterial taxa (indicated as operational taxonomic unit, OTU) and the cyanotoxin. The numbers indicate the proportion of all samples where both the OTU and the toxin could be detected. For example, ‘Bound and free MCs’ were present at the same time as OTU_10_1084258 in 88% of all samples. Red color indicates that the correlation between OTU and toxin is positive and blue color if the correlation is negative. The darker the color, the lower the *p*-value (stronger association between OTU and toxin), as calculated according to Spearman’s correlation test. The dendrogram shows how different the OTUs and toxins are, where similarity is measured by Spearman’s correlation between the level of the different bacteria/toxins. A strong correlation was seen between OTU_1 (*Microcystis* PCC 7914) and MCs and between OTU_13732 (*Aphanizomenon* NIES 81) and STX, Figure 3. Both taxa are known to produce MCs and STX, respectively [51].

### 2.5. Morphological Analysis of Cyanobacteria

Morphological analyses of phytoplankton were performed on 103 samples of which 96 had the presence of cyanobacteria. In total, 53 cyanobacterial taxa were found from 19 genera. *Dolichospermum* was the most common genus present in 67 of the samples represented by 12 species of which *D. lemmermannii* was the most common and abundant. *Microcystis* were the second most common genus in the samples present in 63 samples with *M. aeruginosa* as the most abundant species. The third most common genus was *Aphanizomenon* present in 52 of the samples and five species of which *A. gracile* and *A. yezoense* was most abundant. The site with the highest cyanobacterial diversity based on morphological identification was Fjällnorabadet, Lake Trehörningen, Uppland, with 17 taxa identified. In 20 samples, only one cyanobacterial taxon was present, showing blooms with almost a monoculture of cyanobacteria. Cyanobacteria on the genus level from each site is presented in Table S3 (Supplementary Material S2).

## 3. Materials and Methods

### 3.1. Chemicals and Reference Standards

The reference standards of cyanotoxins included in this survey were ordered from several sources (Table S1, Supplementary Material S2). When possible, standards were purchased as solutions. For the substances that were only available as solid standards, stock solutions of 5000 µg/L in methanol were prepared in-house. All stock solutions were stored in the dark at −20 °C. From the stock solutions, three separate standard solution mixtures (A, B, and C) were prepared in methanol (MeOH) at a concentration of 625 µg/L, in which the cyanotoxins were divided between the solutions, as shown in Table S1 (Supplementary Material S2). Solvents used for mobile phase preparation and all other chemicals were of the LC-MS grade, acetonitrile (ACN, Fisher Scientific, Loughborough, UK), methanol (LiChrosolve), and formic acid 98–100% (Merck, Darmstadt, Germany). LC-MS-grade water was purified with Milli-Q purification system (Millipore, Solna, Sweden). An internal standard (IS) solution of deuterium-labeled microcystin LF, D_5_-MC-LF (Gold Standards Diagnostics Horsham Inc., Warminster, PA, USA), was prepared in 100 μg/L concentration by adding 100 μL of D_5_-MC-LF (concentration: 10 μg/mL) to 9900 μL of Milli-Q water/methanol (97% + 3% *v*/*v*). Lugol’s iodine solution (2 g potassium iodide and 1 g iodide in 100 mL distilled water) supplemented with acetic acid was used.

### 3.2. Materials

**Figure 4:**
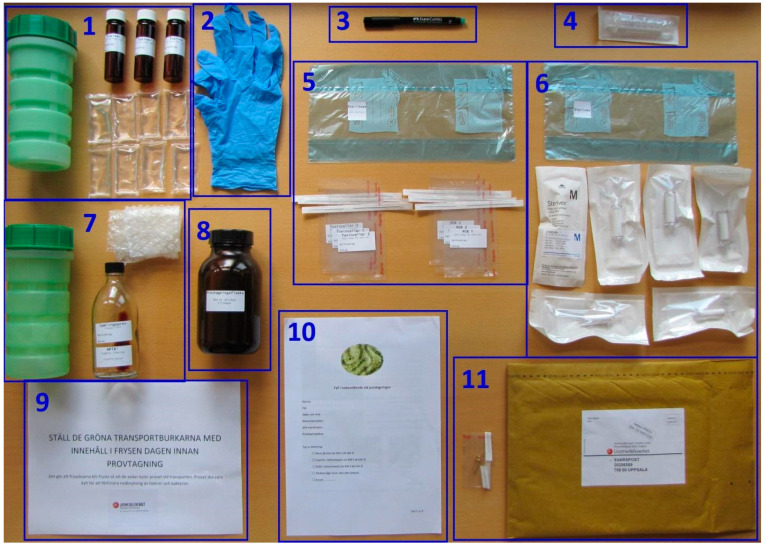
Cyanobloom sampling kit: (1) green transport container with 7–8 freezer bags and three brown filtrate bottles labeled “Toxins 1–3”; (2) a pair of sampling gloves; (3) a permanent marker; (4) a syringe; (5) freezer bag, marked “Steribag/For Sterivex” containing 3 sterile bags marked “Toxin cells 1–3” and 3 sterile bags marked “PCR 1–3”; (6) freezer bag, labeled “Sterivex” containing 6 Sterivex filters; (7) green transport container with “Sample collecting bottle” with 1 mL of Lugol’s solution and bubble wrap for transport; (8) large sampling bottle, amber; (9) sheet with the information to place the kit in the freezer the day before sampling; (10) algal bloom sampling protocol; and (11) padded return envelope with the accessories for closure.

For sampling purposes, a sampling kit was prepared at SFA according to Figure 4, containing a written protocol for sampling and sample handling at the sampling site (Supplementary Material S1). Amber glass bottle with a wide opening (1000 mL) was used as a sampling vessel to take the samples. Polypropylene syringe (Discardit II 10 mL, Beckton Dickinson and Company, Franklin Lakes, NJ, USA) was used for aspiration of a sample from the sampling vessel. Sterivex filter (0.45 μm, MilliPoreSigma, Fisher Scientific GTF AB) was used to filter raw water sample containing cyanobacteria into an amber glass vial (20 mL, Skandinaviska Genetec, Västra Frölunda, Sweden). For further sample preparation in the laboratory, a vortex from Genie 2 Scientific Industries, a Thermo Scientific Haraeus Multi-fuge 3SR+ centrifuge, and an ultrasonication device were used. Mass spectrometry (MS) analyses were performed using a Waters Xevo TQ-S triple quadrupole mass spectrometer coupled to a Waters Acquity UPLC i-Class with a flow through needle sample manager.

### 3.3. Sampling and Sample Preparation

Planktonic bloom samples were collected in June–October 2016 and 2017 (Table 3), from more than one hundred blooms in surface water throughout Sweden including the brackish east coast. The most northern sampling location was at 65.9150371 N, 22.273922 E and the most southern location at 55.4675428 N, 13.4682083 E (distance: 1255 km), as illustrated by Figure 1. An envelope containing sampling material according to Figure 4 was sent by mail to each sampler (e.g., fishermen, private initiatives, water treatment plants, and other voluntary samplers, besides the sampling carried out by the staff at the SFA). The samplers followed a written sampling protocol enclosed in the envelope together with the sampling material. Briefly, on arrival to the sampler, the envelope was placed in a freezer (−20 °C) overnight to prepare the freezer bags for the optimal transport of samples on return to the lab. The principle of the sampling procedure was based on separating cyanobacterial cells from free toxins in the raw water sample using a Sterivex filter, immediately after the sample was taken, in order to ensure that the sample would reflect its toxin profile at the time of sampling. Before the elution of the toxins from the Sterivex filter, containing the retained cyanobacteria, 15 µL of the IS solution (D_5_-MC-LF) was added to the top of the Sterivex filter whereafter 1485 µL of 50% methanol (1:1, MeOH:Milli-Q) was applied using a 10 mL syringe pressure to the Sterivex filter until the entire applied volume was filtered. Lugol’s iodine solution (as described in Section 3.1) was used to preserve the samples for microscopic phytoplankton examination.

#### 3.3.1. Free Toxins

Separation of free cyanotoxins was performed at the sampling site. Samples were taken from three different locations in the cyanobloom by filling a large (1 L) amber glass bottle (the sampling vessel). A portion of the sample was poured into a bottle containing Lugol’s solution to prepare a sample pool of cyanobacterial cells from each of the three sampling locations in the bloom. From the remaining sample volume in the sampling bottle, 20 mL was aspirated with a syringe. The sample volume in the syringe was filtered through a Sterivex filter (with a pore size of 0.45 µm), and the filtrate was collected in a 20 mL amber glass vial. This filtering procedure was applied to each of the three samples taken in the bloom and in each bloom included in this study. At the end of a sampling, at a sampling site, there were six Sterivex filters containing retained sample material, three vials containing filter-eluates with free toxins, and a flask containing a sample pool in Lugol’s solution from the three locations in the bloom. The Sterivex filters containing the retained sample material were packed individually in sterile zip-bags and sent to the lab by mail along with vials containing the filter-eluates and the flask with the sample pool in Lugol’s solution, on the same day as the samples were collected. In addition, the sample package included notes from the sampler with the information regarding sampling date, time, weather and wind, name of the sampling location, GPS coordinates, and the type of bloom collected (e.g., foam on the surface, bloom distributed in the body of water, streaks in the body of water, algae growth from rock/bottom, or additional information about the sample’s look and origin that was not specifically requested in the sampling protocol).

#### 3.3.2. Cell-Bound Toxins

For sample preparation upon sample arrival to the laboratory, the sterile zip-bag with the sample in the Sterivex filter was placed in a freezer at −80 °C. The zip-bag was removed from the freezer after 20 min and thawed in a room-temperate water bath. The freeze–thaw procedure was repeated twice. The Sterivex filter was spiked with 15 μL of D5-microcystin LF directly on top of the filter using an automatic pipette. A 1485 μL volume of 50% methanol in water (1:1 methanol/Milli-Q) was added to the Sterivex filter and filtered using a 10 mL syringe. The filtrate containing cell-bound toxins was collected in an amber LC vial. When the samples were prepared one day before analysis, they were placed in an ultrasonic bath for 5 min prior to analysis. Both free toxins and cell-bound toxins were analyzed using the LC-MS/MS methods described in Section 3.4.

### 3.4. Analysis of Cyanotoxins with LC-MS/MS

Two different LC-MS/MS methods were used, i.e., a reverse-phase (RP, C18) UPLC-MS/MS method for the MCs (18), cylindrospermopsin, nodularin, and anatoxins (2), according to Pekar et al. [2], and an UP-HILIC MS/MS method for the polar saxitoxin and the analogues (ionized in ESI+ and ESI− modes) according to Boundy et al. [38]. All mass spectral analyses were performed using a Waters Xevo TQ-S triple quadrupole mass spectrometer coupled to a Waters Acquity UPLC i-Class with a flow-through needle sample manager. For quantitative analysis, a specific calibration curve was built for each of the toxin analogues using the TargetLynx v 4.1 software (Waters, Milford, MA, USA, 2011).

#### 3.4.1. LC-MS/MS Analysis with C18 Column

For the RP-UPLC-MS/MS analysis, the method published by Pekar et al. [2] was applied without further modifications with respect to the chromatographic separation and the MS-MS detection. This method was accredited in-house for the analysis of cyanotoxins in raw water and drinking water for 22 cyanotoxin congeners. For this study, the sample preparation procedure of the method was extended by introducing the Sterivex filter to allow for a separation of free and cell-bound toxins. A brief description on how the analysis was carried out according to the method is as follows: for the chromatographic separation an ACQUITY BEH C18 UPLC column, 2.1 × 100 mm fitted with a pre-column from VanGuard ACQUITY BEH C18 UPLC, 2.1 × 5 mm, both with a particle size of 1.7 µm (Waters, Manchester, UK). The temperature over the columns was 35 °C during analysis, and the injection volume was 100 µL. Mobile phase A contained 0.1% formic acid (FA) in MilliQ water and mobile phase B 0.1% FA in acetonitrile (ACN). The gradient elution was performed as follows: 0–0.7 min, 2% B, flow 0.3 mL/min; 0.80 min, 2% B, from here the flow started to increase to 0.45 mL/min; 9.0 min, 70% B; 9.1 min, 90% B; 10.0 min, 90% B; 10.1 min, 2% B; and 12.0 min, 2% B. Quantification of cyanotoxins was performed in multiple reaction monitoring (MRM) mode and positive electrospray ionization (ESI+), with a capillary voltage of 3.0 kV. The source offset was 50 V, and the source temperature was 150 °C. Nitrogen (N2) was used as desolvation and cone gas at flows of 650 and 150 L/Hr, respectively. The desolvation gas temperature was 350 °C. The nebulizing gas was also N_2_ at a pressure of 7.0 bars. Argon was used as collision gas at a flow of 0.15 mL/min. The compound-specific MS parameters such as cone voltage (CV), collision energy (CE), and mass transitions (*m*/*z* values) were according to Pekar et al. [2].

#### 3.4.2. LC-MS/MS with HILIC Column

For the UP-HILIC-MS/MS analysis, the method was applied as published by Boundy et al. [38] without further modifications. The extension in sample preparation with the Sterivex filter to separate free from cell-bound toxins was the same as in Section 3.4.1, with the exception that the eluates from the Sterivex filter containing the free or the cell-bound toxins were prepared by diluting the eluate into a diluent of 70% acetonitrile. The chromatographic gradient settings were briefly as follows: mobile phases A1: water/formic acid/NH_4_OH (500:0.075:0.3 *v*/*v*/*v*); B1: acetonitrile/water/formic acid (700:300:0.1 *v*/*v*/*v*); A2: water/formic acid (200:1 *v*/*v*); and B2: Methanol. The initial conditions consisted of 5:95 A1 and B1 at 0.4 mL/min, held for 4 min, thereafter from 5:95 to 50:50 in a linear gradient over 3.5 min. The mobile phase composition was then held while the flow rate was linearly increased to 0.6 mL/min over 1.5 min. The column was then re-equilibrated using a linear gradient to 5:95 with 0.8 mL/min over 0.5 min and then held for 0.6 min. Finally, the flow rate was decreased to 0.4 mL/min and held for 0.4 min. The ionization parameters were as follows: capillary voltage of 3.0 kV, source desolvation temperature of 600 °C, and source ion block temperature of 150 °C. Nitrogen (≥95%) desolation gas flow rate was 1000 L/h, and that of nebulizer gas was 7.00 Bar. The collision gas flow rate of argon was set at 0.15 mL/min. A minimum of two transitions were used for each toxin analogue in the MRM analysis [2,38].

### 3.5. DNA Extraction, Purification, and 16S rRNA Amplicon Sequencing

A total of 70 filter samples were collected in 2016 and 209 filter samples during 2017. The Sterivex filter was opened, and a part of the filter was placed in a PowerBead Tube and disrupted with MP FastPrep^®^-24 using the following settings: speed 6, CY 24 × 2 for 45 s. DNA was then extracted using DNeasy^®^PowerLyzer^®^PowerSoil^®^ Kit from Qiagen according to the manufacturer’s instructions. A two-step PCR method was used to sequence bacteria-specific SSU rRNA amplicon from the DNA samples. The first PCR reaction amplified 570 bp in the variable region V3–V5 [52] of the 16S rRNA gene using primers 357F (CCTACGGGAGGCAGCAG) and 926R (CCGTCAATTCMTTTRAGT). The PCR conditions were as follows: DNA polymerase heat activation at 95 °C (15 min), then 28 cycles containing four steps, i.e., 94 °C (60 s), a step-down to 70 °C (1 s), a ramping rate of 0.4 °C/s to 50 °C (60 s), and a ramping rate of 0.8 °C/s to 72 °C (60 s), this was followed by a final extension at 72 °C (10 min). To account for the random PCR drift [53], the reactions were performed in triplicate. Purification of the PCR products was performed with magnetic AMPure XP beads (Agincourt). PCR products from each sample were pooled and libraries were constructed with index adaptor sequence from the TruSeq DNA LT Sample Prep Kit (Illumina).

### 3.6. Sequence Analysis

Paired-end sequencing (2 × 300 bp) was performed on an Illumina MiSeq (SciLifeLab, Uppsala, Sweden). To remove forward and reverse primer sequences, raw MiSeq run fastq reads were treated with cutadapt [54] and filtered to a MINLENGTH of 100. The 3′ ends were trimmed to a Phred quality score of 10, and forward and reverse reads were merged using VSEARCH [55] v. 1.11.1 with “- -fastq-minovlen” option set to 16. Reads were the de-replicated (- -derep full length) and clustered into centroid OTU’s at a cut-off threshold of 97% using VSEARCH reads. Chimeras were detected and removed using UCHIME [56] with the SILVA123.1_SSUref_tax:99 database [57]. The LCA Classifier [58] 2.0 with the SILVAMOD_106 database was used to assign taxonomy.

### 3.7. Microscopy

Within each cyanobloom site, raw water samples were collected from three locations and mixed to form a pool sample from which a subsample was taken for analysis of phytoplankton, according to the flowchart in Figure 2. Samples were preserved with Lugol’s solution, and cyanobacteria were identified to the finest possible taxon using an inverted light microscope using 100–1000 times of magnification. Phytoplankton were sedimented overnight to a counting slide using an Utermöhl chamber, according to Olrik et al. [40]. Phytoplankton analyses were performed by the certified biodiversity laboratory at the Department of Aquatic Sciences and Assessment, Swedish University of Agricultural Sciences, by highly skilled phytoplankton taxonomists using an array of phytoplankton studies.

### 3.8. Statistical Data Processing

Association between OTUs and cyanotoxins are assessed using Spearman’s correlation and illustrated in a heatmap (Figure 3). Hierarchical clustering using complete linkage and a correlation-based distance measure (sqrt(1-|rho|), where rho is the Spearman correlation coefficient), was used to group OTUs and cyanotoxins.

## 4. Conclusions

This study presents a survey of cyanobacterial toxins and their geographical diversity and distribution in Swedish surface water during cyanobacterial blooms. The geographical scope of the study’s sampling and analysis of cyanotoxins is the largest conducted in Sweden to date. Two different chemical approaches (UPLC-C18-MS/MS and UP-HILIC-MS/MS) were applied to analyze 24 cyanotoxin congeners from 98 sampling sites over a spatial distance of 1255 km. Molecular methods were used to determine cyanobacterial composition and cyanobacterial operational taxonomic unit (OTU) with the highest abundance and prevalence in collected samples. The results from the LC-MS/MS analyses showed an overall high variability in cyanotoxins with MCs as the most commonly occurring cyanotoxins, dominated by MC-RR, followed by STXs as the second most commonly detected group of cyanotoxins. In these toxin groups, the highest toxin concentrations were also measured (at single sampling sites), 26,032 µg/L and 1890.1 µg/L for MCs and STXs, respectively. The detection of saxitoxin analogue decarbamoylsaxitoxin, dcSTX, at four sampling sites in this study is the first report on the presence of dcSTX in surface waters in Sweden. The study further confirms that nodularin belongs in brackish water samples from the Baltic Sea, although it was recently detected in oysters from the west coast of Sweden [35]. Although differences in toxicities of cyanotoxin congeners are known, there are no toxic equivalency factors (TEFs) that uniformly can be applied to adjust for the variation in their activity. Only a few guidelines for cyanotoxin levels exist, and the World Health Organization (WHO) established a TDI of 0.04 µg/kg body weight for chronic exposure to microcystin LR (MC-LR) and recommends a safe limit of 1 µg/L for MC-LR in drinking water [59,60].

The results from the molecular methods show that the OTU with the highest abundance is annotated to *Aphanizomenon* NIES 81, followed by the second most abundant taxon annotated to *Microcystis* PCC 7914.

The sampling approach developed and effectively applied in this study, where the public was interested and engaged in the sample collection, shows the potential of society to contribute to science and knowledge of our waters.

As a result of this study, a handbook has been produced with recommendations for managing risks with cyanotoxins in drinking water [61]. The handbook is aimed for drinking water producers and other drinking water providers, like municipal companies, as well as control authorities. The purpose of the handbook is to be a support in making the necessary decisions in preventing high levels of cyanotoxins from posing a health risk through drinking water consumption.

The results of this study contribute to increased knowledge of the presence and variation of cyanotoxins at studied sampling sites and will be of benefit to tackle future cyanobacterial blooms in fostering water quality for the benefit of public health and the production of drinking water. The data presented could be useful in further research studies on cyanobacterial bloom’s characteristics in Swedish surface waters as well as in research studies in the Scandinavian and the Baltic regions.

## Figures and Tables

**Table 1:** Shares of samples positive for the presence of each cyanotoxin congener identified and quantified as a free form or as the total content in samples. The average and standard deviation (SD) values indicate the distribution in quantities of each toxin congener determined in the LC-MS/MS analysis. The average values presented as lower than the LOQ of the method (0.1 µg/L), and the two decimals represent the sampling sites in which the detected toxin concentration in one or two of three sample replicates was below the LOQ of the method or that the toxin was not detected in either of the three sampling locations in the cyanobloom. * Values for all congeners in the group of MCs and STXs.

Toxin	Share of Positive Samples, Only Free Toxins (%)	Share of Positive Samples (%)	Average Free Toxins (µg/L)	SD Free Toxins (µg/L)	Average Total Quantity (µg/L)	SD Total Quantity (µg/L)
Microcystin RR	19	70	4.9	6.4	262.1	1782.7
Microcystin LR	10	55	148.5	132.1	197.3	682.5
Microcystin WR	1	19	0.06	0.01	6.5	9.7
Microcystin LA	1	4	0.2	0.02	37.1	14.4
Microcystin LY	3	11	2.8	0.8	1.7	1.4
Microcystin LW	1	8	0.6	0.06	1.5	0.7
Microcystin LF	2	8	2.5	0.5	1.2	0.8
Microcystin HtyR	1	13	0	0.01	0.3	0.2
Microcystin HilR	2	16	3.4	0.6	6.9	7.9
Microcystin HtyR [D-Asp3, (E)-Dhb7]	2	9	2.8	0.5	4.9	3.2
Microcystin RR [Dha 7]	20	46	0.7	0.8	6.0	9.6
Microcystin YR	11	36	0.9	0.3	38.1	110.0
Microcystin RR [D-Asp3] + Microcystin RR [D-Asp3, (E)-Dhb7]	10	39	0.5	0.3	5.2	8.5
Microcystin LR [D-Asp3]	3	22	0.4	0.08	14.	17.7
Microcystin (N-methyl-L) R	4	17	1.3	0.4	5.8	6.9
Microcystin HphR [D-Asp3, (E)-Dhb7]	1	10	1.0	0.1	138.7	135.6
Microcystins *	**39**	**82**	**42.0**	**139.1**	**406.9**	**2619.9**
Micropeptin 1106	0	1	0	0	0.1	0.01
Anatoxin-a	1	4	1.8	0.2	2.6	0.6
Homoanatoxin-a	4	6	0.2	0.03	0.2	0.04
Cylindrosper- mopsin	9	10	1.1	0.8	1.1	0.9
Nodularin	8	13	21.7	13.6	155.5	152.7
Decarbamoyl- saxitoxin	7	9	1.6	1.5	4.6	1.9
Saxitoxin	36	45	19.1	188.3	72.9	188.9
Decarbamoyl- neosaxitoxin	0	0	0	0	0	0
Saxitoxins *	**36**	**47**	**19.3**	**189.7**	**72.0**	**190.4**

**Table 2:** Overview of the ten most ubiquitous cyanobacterial taxa across all sampled cyanoblooms using 16S rRNA amplicon analysis combined with SILVAMOD_106 database to assign taxonomy.

Taxonomic Affiliation	OTU Number	Number of Samples with Presence of Each OTU
Nostocales; Nostocaceae; Aphanizomenon NIES 81	OTU_10	243
Synechococcales; Cyanobiaceae; Cyanobium PCC 6307	OTU_13563	222
Nostocales; Microcystaceae; Microcystis PCC 7914	OTU_1	215
Synechococcales; Cyanobiaceae; Cyanobium PCC 6307	OTU_17512	202
Synechococcales; Cyanobiaceae; Cyanobium PCC 6307	OTU_14559	200
Nostocales; Nostocaceae	OTU_933	191
Synechococcales; Cyanobiaceae; Cyanobium PCC 6307	OTU_883	187
Nostocales; Microcystaceae; Snowella 0TU37S04	OTU_9	178
Nostocales; Nostocaceae; Aphanizomenon NIES 81	OTU_13732	156
Nostocales; Microcystaceae; Snowella 0TU37S04	OTU_250	147

**Table 3:** Sampling period and the number of sampling sites positive for one or more of the cyanotoxin analogues included in the study.

Year	Month	Number of Sampling Sites
2016	July	1
	August	10
	September	10
	October	3
2017	June	1
	July	16
	August	44
	September	12
	October	1

## Data Availability

Data are contained within the article.

## Supplementary Materials

The following supporting information can be downloaded at: https://www.mdpi.com/article/10.3390/md22050199/s1, Supplementary Material S1, Sampling protocol; Supplementary Material S2, Table S1: Reference standards of cyanotoxins used in LC-MS/MS analysis. Table S2: Toxin findings in samples from 98 individual sampling sites (cyanoblooms). Table S3: Morphological characterization of cyanobacteria from 98 sampling sites sampled during 2016 and 2017 during bloom conditions. Supplementary Material S3, Table OTUs.
