# Supplementary material for: Cyanotoxin Occurrence and Diversity in 98 Cyanobacterial Blooms from Swedish Lakes and the Baltic Sea
Source: Mar Drugs. 2024 Apr 27;22(5):199. doi: 10.3390/md22050199 (PMC11123207; doi:10.3390/md22050199)
Supplement: Supplementary file 1 [file marinedrugs-22-00199-s001.zip › Supplementary Material 1.pdf]

Livsmedelsverket

Sampling of algal blooms

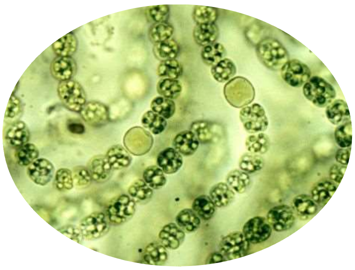

NOTE: Samples must be returned the same day as they were collected.

For questions, contact "Cyanohotline" at phone number **+46 70 311 77 30**, or write to algblomning@slv.se

Sampler:

e-mail address or phone number sampler:

Date:

Time:

Weather & wind:

Address/sampling site:

GPS coordinates:

Type of bloom:

- ☐ **Foam on the surface (see picture 1 on page 2)**
- ☐ **Scattered in the body of water (see picture 2 on page 2)**
- ☐ **Streaks in the body of water (see picture 3 on page 2)**
- ☐ **Algae growth (e.g. rock or bottom)**
- ☐ **Other:………………**

Additional comments:

Video instruction for sampling is available via this link:
https://dreambroker.com/channel/h5s72mgu//oszt8854

Important to know

A bloom is a mass growth of one or more species of cyanobacteria. Blooms often recur seasonally in the same water and benefit from calm, warm weather without rain. They also benefit from a good supply of nutrients, mainly phosphorus and nitrogen.

Blooms occur throughout Sweden, from south to north, and can last from a few hours to several weeks. A bloom is not always visible on the surface, although it can be found several meters down in the body of water or as a growth on the sediment, rock or bottom.

Safety

Since a cyanobacterial bloom can contain toxins in high concentrations**, gloves should be used during the sampling!**

When to sample

Day time. Not during the rain or when it is very windy.

How to recognize a cyanobacterial bloom

Cyanoblooms can look differently. Like:

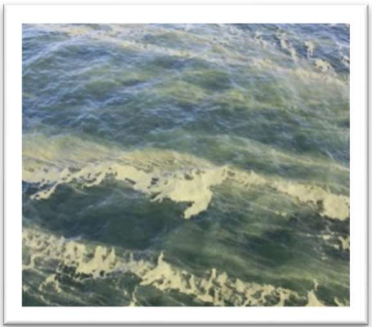

Picture 1: Foam on the surface

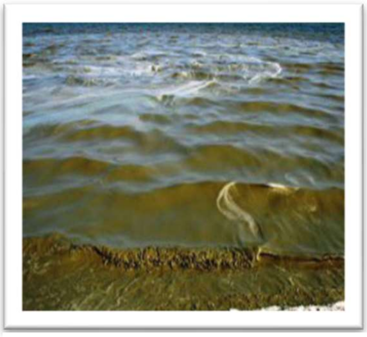

Picture 2: Scattered in the body of water

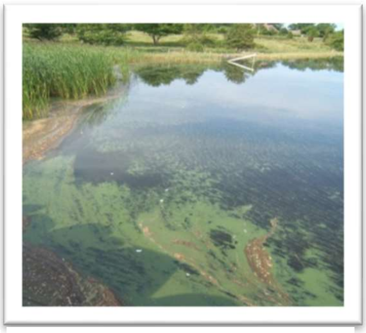

Picture 3: Streaks in the body of water

What to sample

Three water samples should be collected from different places in the cyanobloom, if possible.

Parts of a sampling kit:

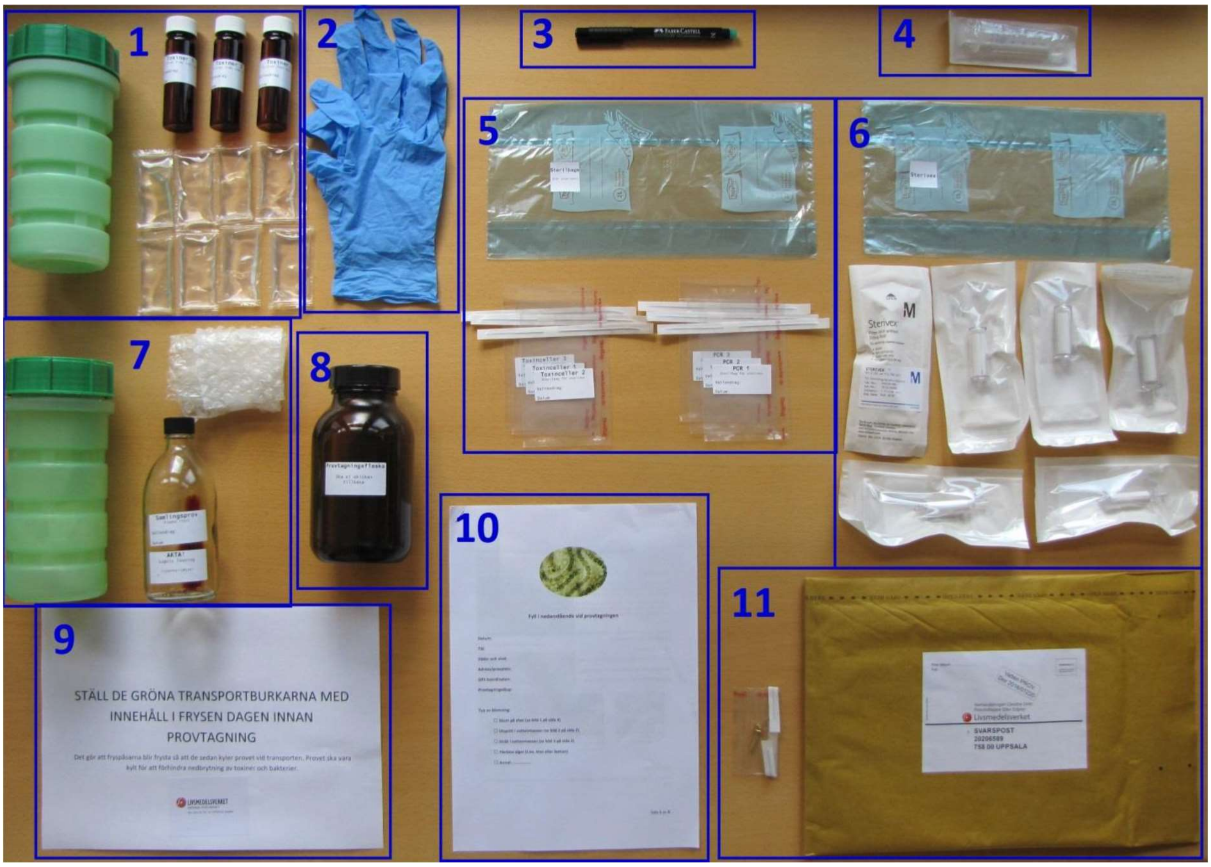

1: Green shipping jar containing:

7-8 freezer bags, three brown filtrate bottles marked ”<u>Toxins 1-3</u>”.

2: A pair of sampling gloves.

3: Pen for writing on labels.

4: Syringe.

5: Freezer bag, marked ”Steribag/For Sterivex” containing:

3 sterile bags, marked "<u>Toxin cells 1-3</u>" and 3 sterile bags, marked "<u>PCR 1-3</u>".

6: Freezer bag, marked ”Sterivex” containing:

6 Sterivex filters.

7: Green shipping jar containing:

”<u>Sample-pool flask</u>” containing 1 mL Lugol´s solution and bubble wrap for shipping.

8: Sampling bottle.

9: Information sheet.

10: Sampling protocol for cyanobloom sample collection.

11: Brown returning-envelope and 2 bag clips.

Sampling is carried out as follows:

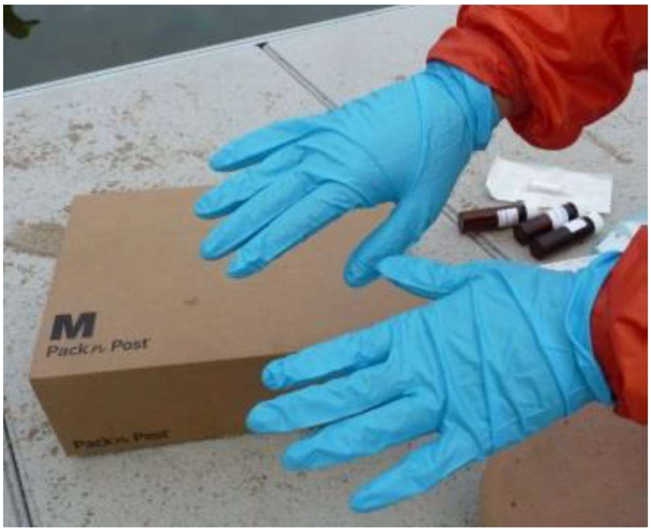

Step 1. **Start by putting the gloves on!**

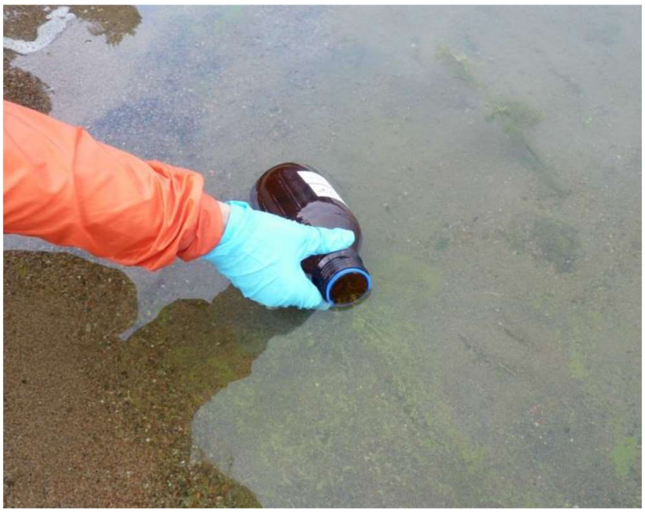

Step 2. Fill the "Sampling Bottle" with water from the bloom. The following (3- 18) steps should be done immediately on site, even if the images are from different locations.

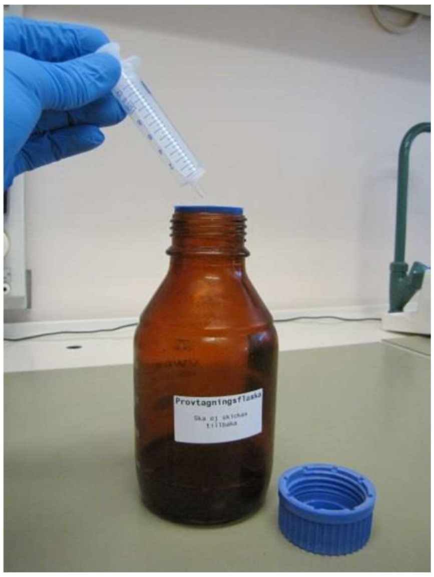

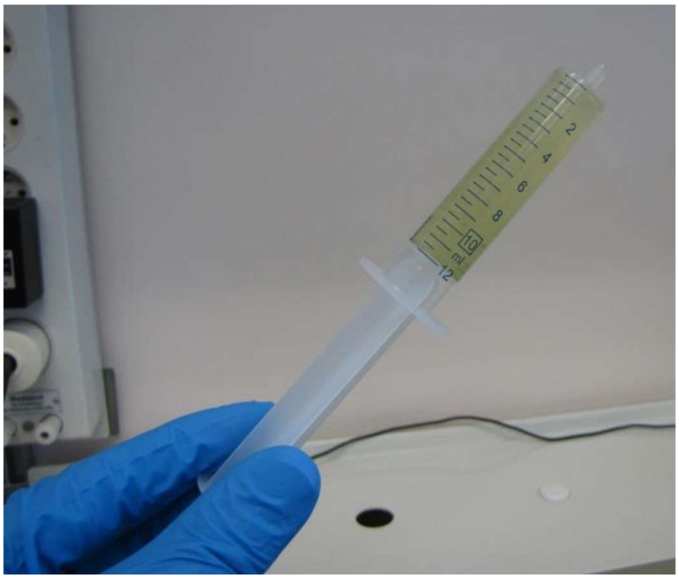

Note: The picture shows a syringe filled with 12 ml of water sample, although 10 ml applies, as stated below.

Step 3. Take a syringe out of the package, shake the "Sampling Bottle" and aspirate 10 ml of water sample from the "Sampling Bottle".

Important! If the cyanobloom is so dense that it is not possible to aspirate the water sample with the syringe or to push 2 ml of liquid through the Sterivex filter, these steps need to be carried out in the lab. In such a situation, the steps 3-16 must be skipped on site near the cyano-bloom. Instead, the filtrate bottles 1, 2 and 3 should be **half-filled** with water from three optional locations in the bloom. Use the water from the same three places in the bloom to fill the "Sample-pool flask" (see step 17). Send the samples back as described earlier (see page 9), with the exception that the Sterivex filters and the sterile bags can be returned as loose parts in the envelope.

Step 4. Take a Sterivex filter out of the package. Take the filtrate bottle labelled "Toxins 1" and unscrew the cap.

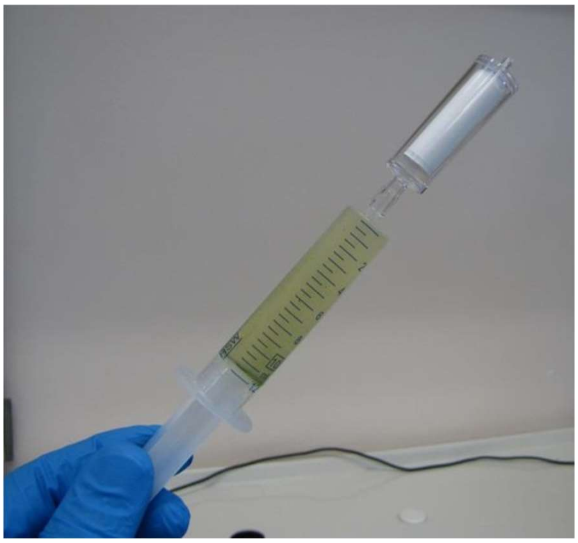
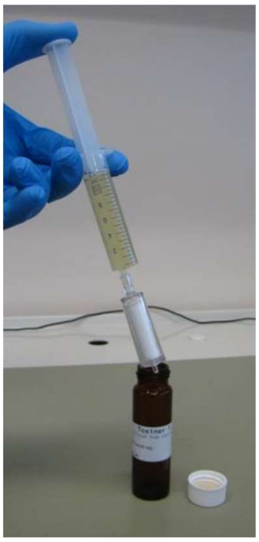

Step 5. Place the Sterivex filter on the syringe (that contains 10 ml of water sample) and place the filtrate bottle "Toxins 1" under the outlet of the Sterivex filter.

Step 6. Push as much water as possible from the syringe through the Sterivex filter and collect the water in the filtrate bottle "Toxins 1".

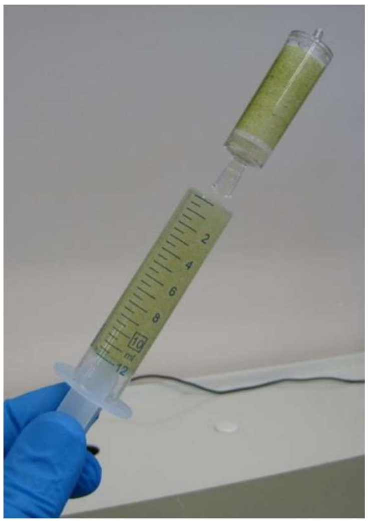
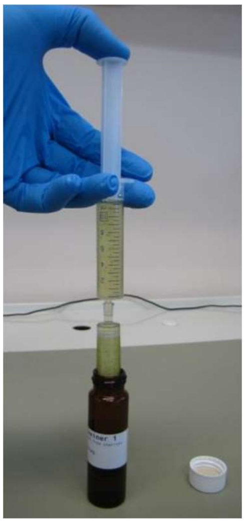
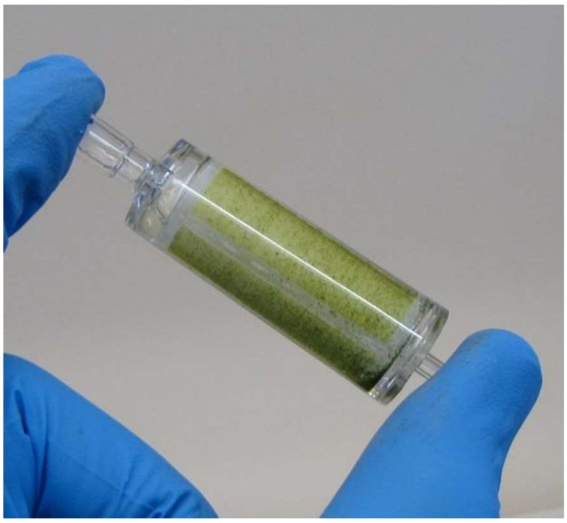

Step 7. If it is not possible to push a total of 10 ml of water sample through the Sterivex filter (as shown in the example picture), a comment on this should be written in the field "volume" including a note on the total volume of water sample that was pushed through the Sterivex filter into the filtrate bottle labeled "Toxins 1". In the picture above, there are 7 ml left in the syringe, meaning that a total of 3 ml of water sample was pushed through the Sterivex filter.

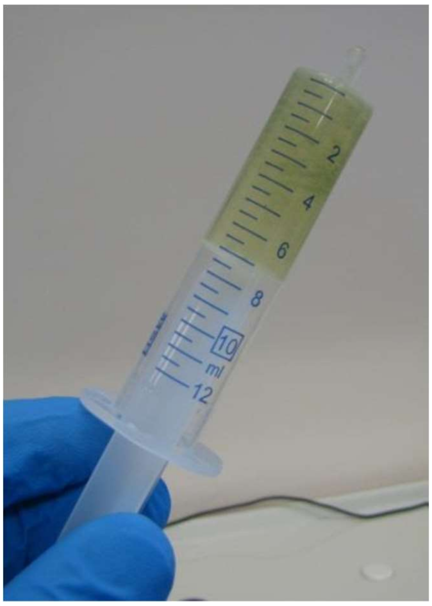

Step 8: Remove the Sterivex filter from the syringe and aspirate air into the syringe, then attach the same Sterivex filter on the syringe again and push the air through the Sterivex filter. Repeat this to get as much liquid as possible into the filtrate bottle labelled "Toxins 1". After this, there should be between 2-10 ml of clear filtrate in the filtrate bottle "Toxins 1".

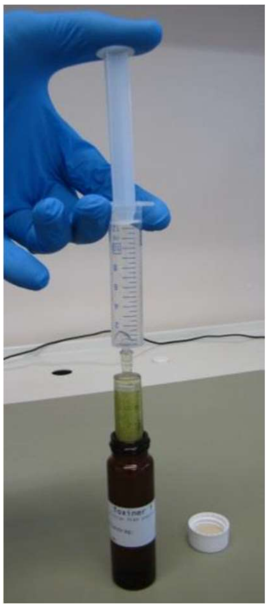 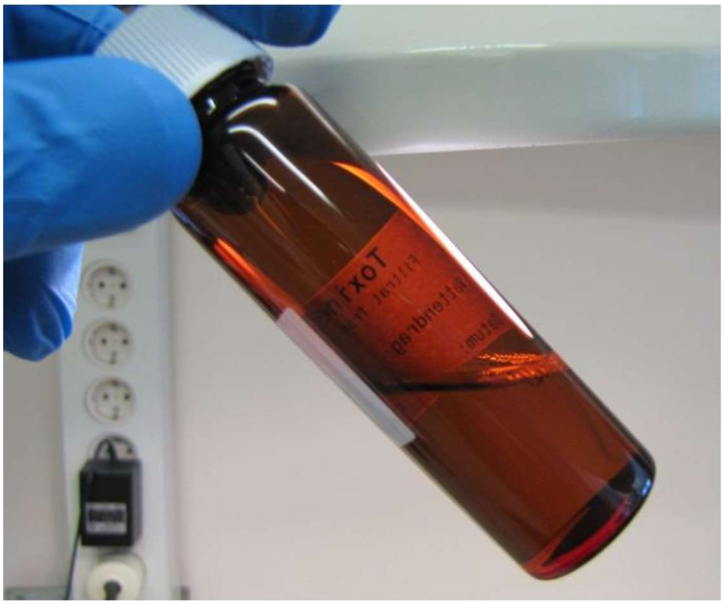

Step 9. Place the Sterivex filter in the sterile bag marked "Toxin cells 1" and put the lid on the filter bottle "Toxins 1". Write down the date and GPS coordinates/sampling site on the labels on both the sterile bag "Toxin cells 1" and the filtrate bottle "Toxins 1".

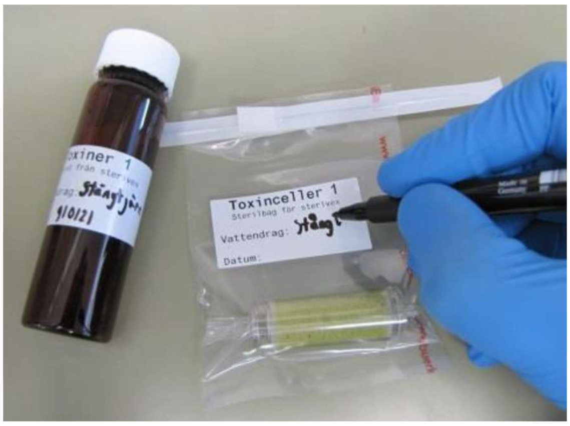

Step 10. Seal the sterile bag labelled "Toxin cells 1" containing the Sterivex filter by pressing as much air as possible out of the sterile bag, roll down the bag sealer and fold the ends.

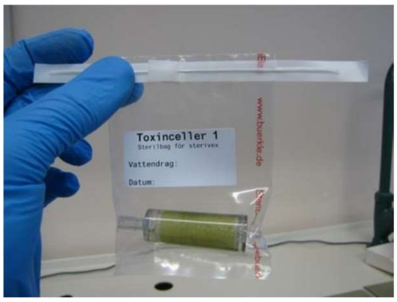

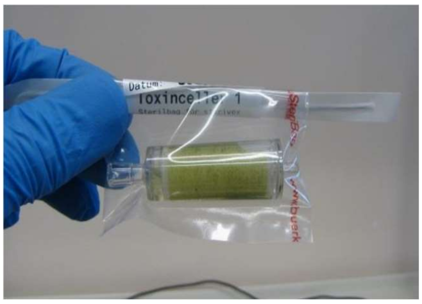 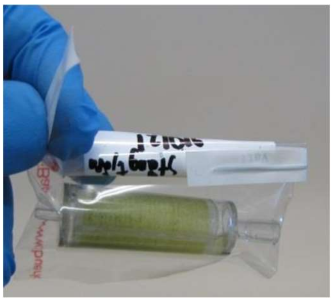

Step 11. Shake the sampling bottle and draw another 10 ml of water up from the sampling bottle with the syringe. The bottle should be shaken so that the cyanobacteria are evenly distributed in the water before the syringe is brought down.

Step 12. Take a new Sterivex filter and filtrate a new portion of water from the sampling bottle. This time, the water that passes through the Sterivex filter does not need to be collected.

Step 13. Remove the Sterivex filter from the syringe and aspirate the air in the syringe. Re-attach the same Sterivex filter and push the air through. Repeat this to remove as much water out of the Sterivex filter as possible.

Step 14. Place the Sterivex filter in a sterile bag labelled "PCR 1". Write down the date and the name of the sampling site. Seal the sterile bag as shown in step 10.

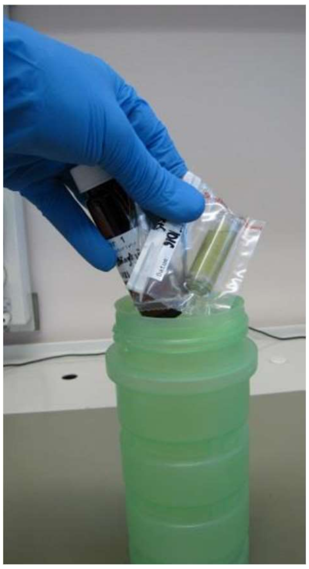

Step 15. Place the filtrate bottle "Toxin cells 1", sterile bag "Toxin cells 1" and sterile bag "PCR 1" in the green shipping jar.

Step 16. Shake sampling bottle again and pour the water from the sampling bottle into the "Sample-pool flask" (containing Lugol's Solution) to the first line.

Step 17. Repeat Step 1 through Step 17 after collecting water from 2 other optional places in the cyanobloom. Then use the filtrate bottles and the sterile bags labelled in the same way as earlier, but with the number 2 and 3 for each of the other two places in the cyanobloom, respectively. At step 16 of the protocol next time, pour the water to the second and third line in the "Sample-pool flask".

Step 18. Place packaging material and other rubbish in one of the freezer bags enclosed the envelope and dispose of the filled freezer bag in waste sorting as combustible.

The following samples are to be placed into the green shipping jars and sent back to the Swedish Food Agency:

Six sterivex filters each in a separate sterile bag (marked "Toxin cells 1, 2, 3 and PCR 1, 2, 3")

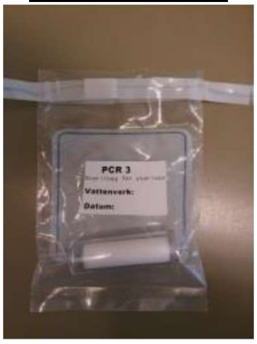

A "Sample-pool flask" (water from 3 different places in the bloom)

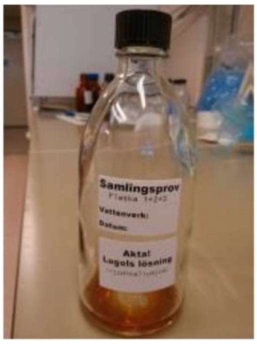

Three filtrate bottles (labeled "Toxins 1", "Toxins 2" and "Toxins 3", filtrate the from Sterivex filter")

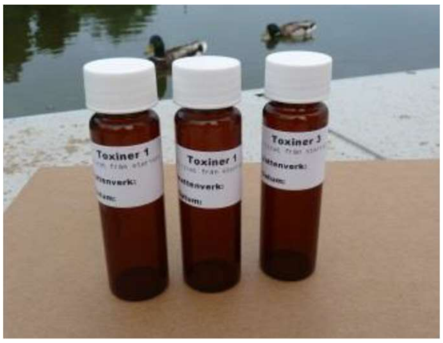

How to organize the samples for mailing

Step 1. Check that the three filtrate bottles are in the green shipping jar with the frozen cooler bags on the bottom.

Step 2. Check that all the sterile bags containing Sterivex filters are in the same shipping jar as the filtrate bottles.

Step 3. The "Sample-pool flask" is placed in the second green shipping jar.

Step 4. The two green shipping jars are placed in the brown envelope together with the filled sampling protocol. Seal the envelope with the clips.

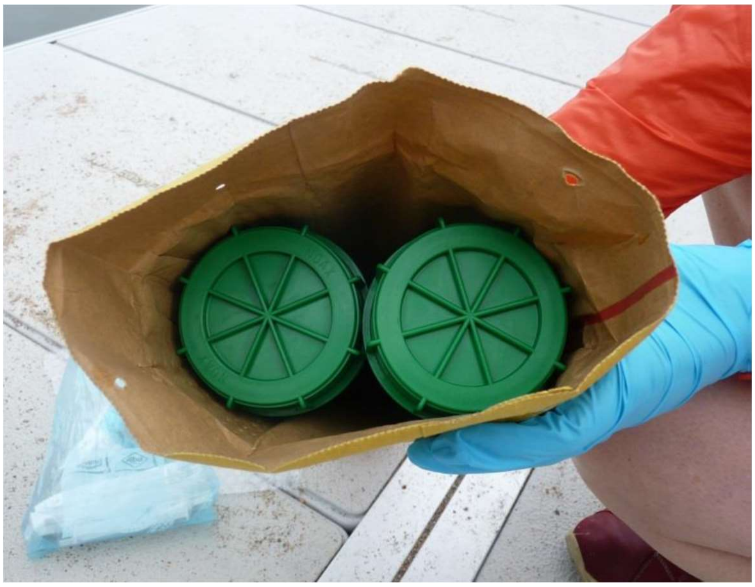

Step 5. Mail the envelope the same day.

Thank You!
