# Supplementary material for: Cyanotoxin Occurrence and Diversity in 98 Cyanobacterial Blooms from Swedish Lakes and the Baltic Sea
Source: Mar Drugs. 2024 Apr 27;22(5):199. doi: 10.3390/md22050199 (PMC11123207; doi:10.3390/md22050199)
Supplement: Supplementary file 1 [file marinedrugs-22-00199-s001.zip › Supplementary material 2.pdf]

marine drugs

Supplementary

Cyanotoxin Occurrence and Diversity in 98 Cyanobacterial Blooms from Swedish Lakes and the Baltic Sea

Caroline Dirks [1,2], Paolo Cappelli [1], Maria Blomqvist [1], Susanne Ekroth [1], Malin Johansson [1], Max Persson [1], Stina Drakare [3], Heidi Pekar [1,4] and Aida Zuberovic Muratovic [1*]

Table S1. Reference standards of cyanotoxins used in LC-MS/MS analysis. a, b Toxins detected in pair. * Toxins in a separate standard mixture used in sample analysis with UP-HILIC-MS/MS method.

Compound name	Standard mix	Source
Anatoxin-a	A	Teknolab Sverige AB
Homoanatoxin-a	A	Teknolab Sverige AB
Cylindrospermopsin	A	Teknolab Sverige AB
Nodularin	A	Biosense Laboratories A/S
Microcystin-RR	A	AH Diagnostics AB
Microcystin-HtyR	A	AH Diagnostics AB
[a]Microcystin-[D-Asp3]-LR	A	AH Diagnostics AB
[b]Microcystin-[D-Asp3]-RR	A	Enzo Life Sciences (Lausen, Switzerland)
Microcystin-LA	B	AH Diagnostics AB
Microcystin-LF	B	Teknolab Sverige AB
Microcystin-LW	B	Teknolab Sverige AB
Microcystin-LY	B	AH Diagnostics AB
Microcystin-YR	B	Teknolab Sverige AB
Microcystin-LR	B	AH Diagnostics AB
Microcystin-WR	C	AH Diagnostics AB
Microcystin-HilR	C	AH Diagnostics AB
Microcystin-(N-methyl-L)-R	C	Marbionc (Wilmington, NC, USA)
Microcystin-[D-Asp3, (E)-Dhb7]-HphR	C	Marbionc (Wilmington, NC, USA)
Microcystin-[D-Asp3, (E)-Dhb7]-HtyR	C	Marbionc (Wilmington, NC, USA)
[a]Microcystin-[Dha7]-LR	C	Teknolab Sverige AB
[b]Microcystin-[D-Asp3, (E)-Dhb7]-RR	C	Teknolab Sverige AB
Saxitoxin	*	National Research Council, Halifax, Canada
Decarbamoylsaxitoxin	*	National Research Council, Halifax, Canada
Decarbamoylneosaxitoxin	*	National Research Council, Halifax, Canada

Table S2. Toxin findings in samples from 98 individual sampling sites (cyanoblooms). Each value represents toxin´s average quantity in µg/L of toxin contents in samples taken at three separate locations in a cyanobloom. In cases where toxins were detected below the LOQ of the method (0.1 µg/L) for all three locations are presented as <LOQ for the entire cyanobloom. In situations where toxins could be quantified above the LOQ of the method in one or two out of three locations at the same sampling site the average of the toxin concentrations was calculated applying "0" for the locations in which the toxin was not detected. In this way exclusion of single positive sample replicates of a cyanobloom that are still relevant to visualize in the study was avoided and a more representative toxin concentration data was obtained. Consequently, some of the average quantities fell below the LOQ of the method, which are presented with two decimals in blue. Right-hand column shows the toxin groups detected at each of the sampling sites. hATX was detected at five sampling sites and always at levels ≤LOQ of the method. *, no toxins were detected at LOQ≥0.1 µg/L for the method at any of the three sampling locations of the cyanobloom. na, sample is not analysed for this toxin.

Sample name	Sampling site (cyanobloom)	Sampling Date	MCs (free)	MCs (tot)	NOD (free)	NOD (tot)	ATXs (free)	ATXs (tot)	CYN (free)	CYN (tot)	STXs (free)	STXs (tot)	Toxin groups identified in sample
BS1	Badplatsstämmorna, Mullsjön, Västergötland	2016-09-01	0.03	0.2	<LOQ	<LOQ	<LOQ	<LOQ	<LOQ	<LOQ	na	<LOQ	MCs
BR1	Bastås råvattenpumpstation, Arboga	2016-09-20	<LOQ	2.4	<LOQ	<LOQ	<LOQ	0.1	0.07	0.2	na	<LOQ	MCs, ATXs, hATX, CYN
ES1	Edasjön, Uppland	2016-08-24	na	0.3	<LOQ	<LOQ	<LOQ	<LOQ	<LOQ	<LOQ	na	<LOQ	MCs
FnB1	Fjällnorabadet, Trehörningen, Uppland	2016-08-24	na	0.8	<LOQ	<LOQ	<LOQ	<LOQ	<LOQ	<LOQ	na	1.3	MCs, STXs
FS1	Funbosjön, Uppland	2016-08-24	na	0.3	<LOQ	<LOQ	<LOQ	<LOQ	<LOQ	<LOQ	na	<LOQ	MCs
GåV1	Gumåsviken, Kramfors	2016-10-17	<LOQ	0.6	<LOQ	<LOQ	<LOQ	<LOQ	<LOQ	<LOQ	na	<LOQ	MCs
GV1	Görväln, Mälaren	2016-10-17	<LOQ	0.1	<LOQ	<LOQ	<LOQ	<LOQ	<LOQ	<LOQ	na	<LOQ	MCs
KS1	Kottalsjön, Lidingö	2016-09-09	na	28.6	<LOQ	<LOQ	<LOQ	<LOQ	<LOQ	<LOQ	na	<LOQ	MCs
KH1	Kråkö hamn, Hälsingland	2016-08-24	na	6.0	<LOQ	285.0	<LOQ	1.5	<LOQ	<LOQ	na	<LOQ	MCs, NOD, ATXs
LT1	Lojsta träsk, Gotland	2016-07-27	<LOQ	0.2	<LOQ	<LOQ	<LOQ	<LOQ	<LOQ	<LOQ	na	1.0	MCs, STXs
LäB1	Lyssnaängsbadet, Ekoln, Uppland	2016-08-24	na	0.4	<LOQ	<LOQ	<LOQ	<LOQ	<LOQ	<LOQ	na	<LOQ	MCs
Lu1	Lövudden, Mälaren	2016-09-25	0.2	37.1	<LOQ	<LOQ	<LOQ	<LOQ	<LOQ	<LOQ	na	0.3	MCs, STXs
Fnp 1	Pålsund, Trehörningen, Uppland	2016-08-24	na	4.6	<LOQ	<LOQ	<LOQ	<LOQ	<LOQ	<LOQ	na	10.5	MCs, STXs
GL1	Gravlången, Trollhättan	2016-09-21	<LOQ	3.8	<LOQ	<LOQ	<LOQ	<LOQ	<LOQ	<LOQ	na	<LOQ	MCs
Su1	Stenungssund, Kraftmarksvägen 44432	2016-10-14	<LOQ	6.8	<LOQ	<LOQ	<LOQ	<LOQ	<LOQ	<LOQ	na	6.2	MCs, STXs
SH1	Storahästefjorden, Rotenäs 114	2016-09-20	0.1	10.2	<LOQ	<LOQ	<LOQ	<LOQ	<LOQ	<LOQ	na	<LOQ	MCs
St1	Sundstatjärn	2016-09-07	0.03	0.2	<LOQ	<LOQ	<LOQ	<LOQ	<LOQ	<LOQ	na	23.0	MCs, STXs

SB1	Sunnerstabadet, Ekoln, Uppland	2016-08-24	na	1.1	<LOQ	<LOQ	<LOQ	<LOQ	<LOQ	<LOQ	na	<LOQ	MCs
SD1	Svandammen, Uppland	2016-09-26	na	0.4	<LOQ	<LOQ	<LOQ	<LOQ	<LOQ	<LOQ	na	<LOQ	MCs
Så1	Svartån, Västerås, Västmanland	2016-09-27	na	26032.0	na	na	na	4.2	na	na	na	3.1	MCs, ATXs, STXs
Hå1	Utanför Lomma/Höjeån, Skåne	2016-08-28	<LOQ	<LOQ	<LOQ	<LOQ	<LOQ	<LOQ	<LOQ	<LOQ	na	11.9	STXs
VB1	Vombsjön, båtplatsen, Skåne	2016-08-31	7.3	63.9	<LOQ	<LOQ	<LOQ	<LOQ	<LOQ	<LOQ	na	0.2	MCs, STXs
VS1	Vombsjön, Västra sidan	2016-09-20	0.03	10.5	<LOQ	<LOQ	<LOQ	<LOQ	<LOQ	<LOQ	na	<LOQ	MCs
VSB1	Vårdsätrabadet, Ekoln, Uppland	2016-08-28	na	556.0	<LOQ	<LOQ	<LOQ	<LOQ	<LOQ	<LOQ	na	<LOQ	MCs
17-02	Stenholmen, Dalarö	2017-06-23	<LOQ	<LOQ	<LOQ	1.1	<LOQ	<LOQ	<LOQ	<LOQ	<LOQ	<LOQ	NOD
17-03	Hammarskogsbadet, Ekoln, Uppland	2017-07-12	0.5	1.6	<LOQ	<LOQ	<LOQ	<LOQ	<LOQ	<LOQ	<LOQ	<LOQ	MCs
17-04	Rännavägen, Gävle	2017-07-11	1.0	1.06	<LOQ	<LOQ	<LOQ	<LOQ	<LOQ	<LOQ	<LOQ	<LOQ	MCs
17-05	Krönbadet, Vimmerby	2017-07-13	0.4	1.7	<LOQ	<LOQ	<LOQ	<LOQ	<LOQ	<LOQ	<LOQ	<LOQ	MCs
17-06	Ringsjön, Östra, Gamlabo	2017-07-13	<LOQ	7.3	<LOQ	<LOQ	<LOQ	<LOQ	<LOQ	<LOQ	<LOQ	<LOQ	MCs
17-07	Rörbacks Hamn	2017-07-12	2.9	2.9	<LOQ	<LOQ	<LOQ	<LOQ	<LOQ	<LOQ	<LOQ	<LOQ	MCs
17-08	Herrö Pålsbo, Svegssjön, Sveg	2017-07-15	<LOQ	<LOQ	<LOQ	<LOQ	<LOQ	<LOQ	<LOQ	<LOQ	1.7	<LOQ	STXs
17-09	Strömsborgsvägen 72, Norrtälje	2017-07-20	0.2	0.4	4.1	16.1	<LOQ	<LOQ	<LOQ	<LOQ	0.1	0.2	MCs, NOD, STXs
17-10	Hargsjön, Mjölby	2017-07-20	<LOQ	2.4	<LOQ	<LOQ	<LOQ	<LOQ	<LOQ	<LOQ	0.4	0.6	MCs, STXs
17-11	Svegsjön, Herrö	2017-07-20	<LOQ	<LOQ	<LOQ	<LOQ	0.2	0.2	<LOQ	<LOQ	2.8	4.5	ATXs, hATX, STXs
17-12	Ruggen, Åkersberga	2017-07-23	0.41	6.4	<LOQ	<LOQ	<LOQ	<LOQ	<LOQ	<LOQ	1.6	3.2	MCs, STXs
17-13	Dämstasjön, Kramfors	2017-07-26	<LOQ	<LOQ	<LOQ	<LOQ	<LOQ	<LOQ	<LOQ	<LOQ	0.1	0.3	STXs
17-14	Bergsbadet, Linköping	2017-07-28	<LOQ	0.4	<LOQ	<LOQ	<LOQ	<LOQ	<LOQ	<LOQ	14.7	14.7	MCs, STXs
17-15	Sandvik, Linköping	2017-07-28	<LOQ	0.2	<LOQ	<LOQ	<LOQ	<LOQ	<LOQ	<LOQ	<LOQ	<LOQ	MCs
17-16	Granön, Båtskärsnäs	2017-07-27	0.2	0.3	<LOQ	<LOQ	<LOQ	<LOQ	<LOQ	<LOQ	11.3	12.1	MCs, STXs, dcSTX
17-17	Staka kraftverk, Flian	2017-07-28	172.5	301.3	<LOQ	<LOQ	0.2	0.2	<LOQ	<LOQ	<LOQ	<LOQ	MCs, ATXs, hATX
17-18	Gamla Lövöns kraftverk, Fångsjön	2017-07-26	na	<LOQ	na	<LOQ	na	<LOQ	na	<LOQ	1.0	1.9	STXs
17-19	Hälsjön, Alafors	2017-08-02	<LOQ	5.3	<LOQ	<LOQ	<LOQ	<LOQ	<LOQ	<LOQ	<LOQ	<LOQ	MCs
17-20	Vimmesjöns badplats, Alafors	2017-08-02	<LOQ	0.3	<LOQ	<LOQ	<LOQ	<LOQ	<LOQ	<LOQ	<LOQ	<LOQ	MCs
17-21	Sjon Anten, Alsingsås	2017-08-01	<LOQ	<LOQ	<LOQ	<LOQ	<LOQ	<LOQ	<LOQ	<LOQ	37.5	62.7	STXs

17-22	Rånnavägs badplats, Surströmmasjön, Ulricehamn	2017-08-03	<LOQ	37.5	<LOQ	<LOQ	<LOQ	<LOQ	<LOQ	<LOQ	223.7	424.4	MCs, STXs, dcSTX
17-23	Lofthammar, Västervik	2017-08-04	1382.1	5119.0	<LOQ	<LOQ	<LOQ	<LOQ	<LOQ	<LOQ	<LOQ	<LOQ	MCs
17-24	Svanbergabadet, Norrtälje (Erken)	2017-08-08	0.1	1.2	<LOQ	<LOQ	<LOQ	<LOQ	<LOQ	<LOQ	<LOQ	<LOQ	MCs
17-25	Råvattenintaget till Rottne Vattenverk	2017-08-08	<LOQ	<LOQ	<LOQ	<LOQ	<LOQ	<LOQ	<LOQ	<LOQ	<LOQ	<LOQ	<LOQ
17-26	Öved, Vombsjön	2017-08-07	<LOQ	1.2	<LOQ	<LOQ	<LOQ	<LOQ	<LOQ	<LOQ	<LOQ	<LOQ	MCs
17-27	Lammhults vattenverk, råvattenintaget, Stora Värmen	2017-08-08	<LOQ	0.3	<LOQ	<LOQ	<LOQ	<LOQ	<LOQ	<LOQ	<LOQ	<LOQ	MCs
17-28	Ågesta, Stockholm, Magelungen	2017-08-08	0.5	1.3	<LOQ	<LOQ	1.8	1.8	<LOQ	<LOQ	<LOQ	<LOQ	MCs, ATXs
17-29	Svinstadsjöns badplats, Linköping	2017-08-09	<LOQ	4.5	<LOQ	<LOQ	<LOQ	<LOQ	<LOQ	<LOQ	<LOQ	<LOQ	MCs
17-30	Glaningen sjö, Ludvika	2017-08-09	na	<LOQ	na	<LOQ	na	<LOQ	na	<LOQ	<LOQ	<LOQ	*No toxins quantities ≥ LOQ
17-31	Växjösjön, Växjö	2017-08-14	<LOQ	0.3	<LOQ	<LOQ	<LOQ	<LOQ	<LOQ	<LOQ	<LOQ	<LOQ	MCs
17-32	Södra Bergundasjön	2017-08-14	<LOQ	11.0	<LOQ	<LOQ	<LOQ	<LOQ	<LOQ	<LOQ	<LOQ	<LOQ	MCs
17-33	Övden, Alvesta kommun	2017-08-08	0.3	2.2	<LOQ	<LOQ	<LOQ	<LOQ	<LOQ	<LOQ	1642.3	1891.0	MCs, STXs, dcSTX
17-34	Trummen, Vaxsjö	2017-08-14	<LOQ	0.4	<LOQ	<LOQ	<LOQ	<LOQ	<LOQ	<LOQ	<LOQ	<LOQ	MCs
17-35	Tisarens utlopp, Hallsberg	2017-08-15	<LOQ	2.1	<LOQ	<LOQ	<LOQ	<LOQ	<LOQ	<LOQ	0.04	0.08	MCs, STXs
17-36	Virens utlopp, Katrineholm	2017-08-15	0.3	1.9	<LOQ	<LOQ	<LOQ	<LOQ	<LOQ	<LOQ	0.06	0.1	MCs, STXs
17-37	Ellestadssjön, Sjöbo	2017-08-16	<LOQ	1.4	<LOQ	<LOQ	<LOQ	<LOQ	8.2	9.5	4.4	8.8	MCs, CYN, STXs, dcSTX
17-38	Dånviken, Rönninge	2017-08-17	0.1	41.8	<LOQ	<LOQ	0.1	0.1	<LOQ	<LOQ	2.5	4.9	MCs, ATXs, hATX, STXs
17-39	Yngaren, Nyköping	2017-08-17	0.5	222.4	<LOQ	<LOQ	<LOQ	<LOQ	<LOQ	<LOQ	<LOQ	<LOQ	MCs
17-40	Runnviken	2017-08-17	<LOQ	0.7	<LOQ	<LOQ	<LOQ	<LOQ	<LOQ	<LOQ	<LOQ	<LOQ	MCs
17-41	Stehags badplats, Västra Ringsjön	2017-08-18	<LOQ	0.7	<LOQ	<LOQ	<LOQ	<LOQ	<LOQ	<LOQ	<LOQ	<LOQ	MCs
17-42	Roxen, Linköping	2017-08-18	<LOQ	<LOQ	<LOQ	<LOQ	<LOQ	<LOQ	<LOQ	<LOQ	6.7	7.6	STXs
17-43	Regnaren, Nyköping	2017-08-22	0.07	0.6	<LOQ	<LOQ	<LOQ	<LOQ	<LOQ	<LOQ	1.7	3.4	MCs, STXs
17-44	Börringesjön, Svedala	2017-08-22	<LOQ	0.2	<LOQ	<LOQ	<LOQ	<LOQ	0.04	0.07	0.8	1.7	MCs, CYN, STXs
17-45	Finjasjön, Skåne	2017-08-22	<LOQ	0.5	<LOQ	<LOQ	<LOQ	<LOQ	<LOQ	<LOQ	<LOQ	<LOQ	MCs
17-46	Hallbosjön, Nyköping	2017-08-22	<LOQ	0.6	<LOQ	<LOQ	<LOQ	<LOQ	0.1	0.1	<LOQ	<LOQ	MCs, CYN

17-47	Fjällfotasjön	2017-08-22	<LOQ	0.7	<LOQ	<LOQ	<LOQ	<LOQ	0.4	0.4	0.04	0.08	MCs, CYN, STXs
17-48	Arkelstorpsviken, Skåne	2017-08-22	<LOQ	2.9	<LOQ	<LOQ	<LOQ	<LOQ	<LOQ	<LOQ	<LOQ	<LOQ	MCs
17-49	Hornsundssjön, Flen	2017-08-22	0.1	18.2	<LOQ	<LOQ	<LOQ	<LOQ	<LOQ	<LOQ	<LOQ	<LOQ	MCs
17-50	Hjälmaren	2017-08-22	<LOQ	<LOQ	<LOQ	<LOQ	<LOQ	<LOQ	<LOQ	<LOQ	<LOQ	<LOQ	*No toxins quantities ≥ LOQ
17-51	Vombsjön	2017-08-23	<LOQ	8.3	<LOQ	<LOQ	<LOQ	<LOQ	<LOQ	<LOQ	<LOQ	<LOQ	MCs
17-52	Väsbyviken, Torshälla	2017-08-22	<LOQ	0.9	<LOQ	<LOQ	<LOQ	<LOQ	<LOQ	<LOQ	<LOQ	<LOQ	MCs
17-53	Västeråsfjärden, Mälaren	2017-08-23	0.5	37.8	<LOQ	<LOQ	<LOQ	<LOQ	<LOQ	<LOQ	<LOQ	<LOQ	MCs
17-54	Havgårdssjön, Dävelstorp	2017-08-23	<LOQ	21.4	<LOQ	<LOQ	<LOQ	<LOQ	<LOQ	<LOQ	6.5	9.7	MCs, STXs
17-55	Krageholmssjön rastplats (Hästhagsv.)	2017-08-23	8.4	88.4	<LOQ	<LOQ	<LOQ	<LOQ	0.1	0.2	0.03	0.05	MCs, CYN, STXs
17-56	Näsbyholmssjön, Skåne	2017-08-23	<LOQ	<LOQ	<LOQ	<LOQ	<LOQ	<LOQ	<LOQ	<LOQ	<LOQ	<LOQ	*No toxins quantities ≥ LOQ
17-57	Östra Ringsjön, Skåne	2017-08-23	<LOQ	0.7	<LOQ	<LOQ	<LOQ	<LOQ	<LOQ	<LOQ	<LOQ	<LOQ	MCs
17-58	Råbelövssjön, Ekestad	2017-08-22	0.3	42.1	<LOQ	<LOQ	<LOQ	<LOQ	0.2	0.2	0.7	1.5	MCs, CYN, STXs
17-59	Vadsbrosjön, Nyköping	2017-08-23	1.1	149.5	<LOQ	<LOQ	<LOQ	<LOQ	<LOQ	<LOQ	2.4	4.7	MCs, STXs
17-60	Fält Danviken, Salems kommun	2017-08-24	52.1	352.8	<LOQ	<LOQ	<LOQ	<LOQ	<LOQ	<LOQ	<LOQ	<LOQ	MCs
17-62	Danviken, Salems kommun	2017-09-02	1.3	51.8	<LOQ	<LOQ	<LOQ	<LOQ	<LOQ	<LOQ	<LOQ	<LOQ	MCs
17-63	Storsjön, Gåsnäs, Kramfors	2017-09-04	<LOQ	2.2	<LOQ	<LOQ	<LOQ	<LOQ	<LOQ	<LOQ	<LOQ	<LOQ	MCs
17-64	Hjorten, Västervik	2017-08-28	0.5	4.9	<LOQ	<LOQ	<LOQ	<LOQ	<LOQ	<LOQ	<LOQ	<LOQ	MCs
17-65	Hjorten, Västervik	2017-08-31	0.7	1.1	<LOQ	<LOQ	<LOQ	<LOQ	<LOQ	<LOQ	<LOQ	<LOQ	MCs
17-66	Vombsjön, Östra Vombs badplats, Skåne	2017-09-06	0.2	4.5	<LOQ	<LOQ	<LOQ	<LOQ	<LOQ	<LOQ	<LOQ	<LOQ	MCs
17-67	Finjasjön, Björkviken, Tormstorp	2017-09-06	0.5	4.9	<LOQ	<LOQ	<LOQ	<LOQ	<LOQ	<LOQ	1.0	2.0	MCs, STXs
17-68	Östra Ringsjön, Skåne	2017-09-06	0.1	1.7	<LOQ	<LOQ	<LOQ	<LOQ	0.09	0.3	<LOQ	<LOQ	MCs, CYN
17-69	Ringsjö	2017-09-11	0.10	2.0	<LOQ	<LOQ	<LOQ	0.15	<LOQ	<LOQ	<LOQ	<LOQ	MCs, ATXs
17-70	Långhalsen	2017-09-11	<LOQ	0.4	<LOQ	<LOQ	<LOQ	<LOQ	0.2	0.2	<LOQ	<LOQ	MCs, CYN
17-71	Dånviken, Rönningen	2017-09-08	1.6	127.3	<LOQ	<LOQ	0.2	0.2	<LOQ	<LOQ	<LOQ	<LOQ	MCs, ATXs, hATX
17-72	Västerås gästhamn	2017-09-13	0.20	244.0	<LOQ	<LOQ	<LOQ	<LOQ	<LOQ	<LOQ	<LOQ	<LOQ	MCs
17-73	Linnéasjön, Nybro	2017-09-18	<LOQ	<LOQ	<LOQ	<LOQ	<LOQ	<LOQ	<LOQ	<LOQ	0.6	1.2	STXs
17-74	Sövdesjön, Sjöbo	2017-09-18	0.7	56.5	<LOQ	<LOQ	<LOQ	<LOQ	<LOQ	<LOQ	<LOQ	<LOQ	MCs

17-75	Fiskekroken, Härnösand	2017-09-28	<LOQ	<LOQ	132.6	1514.2	<LOQ	3.0	<LOQ	<LOQ	0.6	1.2	NOD, ATXs, STXs
17-76	Sträsundsviken, Köpmannaholmen	2017-10-02	<LOQ	<LOQ	<LOQ	1.4	<LOQ	<LOQ	<LOQ	<LOQ	<LOQ	<LOQ	NOD

Table S3. Morphological characterization of cyanobacteria from 98 sampling sites sampled during 2016 and 2017 during bloom conditions. For simplification only genera level is shown. Genera names are sorted based on how many samples there where present in. Relative abundance based on phytoplankton counts were used to categorize into groups of dominance. +++, high abundance (>50%); ++, medium abundance (1-50%); + low abundance (<1%); and na (in Comment), sample not available for morphological identification of cyanobacteria.

Sample name	Sampling site	Sampling Date	Dolichospermum	Microcystis	Aphanizomenon	Woronichinia	Planktolyngbya	Planktothrix	Cuspidothrix	Limnothrix	Gloeotrichia	Pseudanabaena	Snowella	Chroococcus	Nodularia	Radiocystis	Anabaenopsis	Merismopedia	Geitlerinema	Oscillatoria	Romeria	Comment
BS1	Badplatsstämmorna, Mullsjön, Västergötaland	2016-09-01		++		+++							++									No cyanobacterial dominance. High abundance of green algae and diatoms.
BR1	Bastås råvattenpumpstation, Arboga	2016-09-20	++		++		++			+++		++										High abundance of diatoms
ES1	Edasjön, Uppland	2016-08-24	++		+++	++	++	++														Total dominance of cyano-bacteria.
FnB1	Fjällnorabadet, Trehörningen, Uppland	2016-08-24	+++	++		++	++			++		++										
FS1	Funbosjön, Uppland	2016-08-24	+++	++																		
GåV1	Gumåsviken, Kramfors	2016-10-17						+++														Also Closterium, a desmid
GV1	Görväln, Mälaren	2016-10-17		+	+			++		+		+++										Also several diatoms and cryptophytes
KS1	Kottalasjön, Lidingö	2016-09-09	++	++	+	+++			+													
KH1	Kråkö hamn, Hälsingland	2016-08-24																				na
LT1	Lojsta träsk, Gotland	2016-07-27	+	+																		Almost no cyanobacteria
LäB1	Lyssnaängsbadet, Ekoln, Uppland	2016-08-24	++	++						+++		++									++	No dominance of cyanobacteria. More diatoms and cryptophytes.

Sample name	Sampling site	Sampling Date	Dolichospermum	Microcystis	Aphanizomenon	Woronichinia	Planktolyngbya	Planktothrix	Cuspidothrix	Limnothrix	Gloeotrichia	Pseudanabaena	Snowella	Chroococcus	Nodularia	Radiocystis	Anabaenopsis	Merismopedia	Geitlerinema	Oscillatoria	Romeria	Comment
Lu1	Lövudden, Mälaren	2016-09-25	++	+++	++	++																
Fnp 1	Pålsund, Trehörningen, Uppland	2016-08-24	+++	++	++																	
GL1	Gravlången, Trollhättan	2016-09-21	+++	++		++																
Su1	Stenungssund, Kraftmarksvägen 44432	2016-10-14				+++																
SH1	Storahästefjorden, Rotenäs 114	2016-09-20	+	++	+++	++																
St1	Sundstatjärn	2016-09-07	+++			++							++									More desmids than cyanobacteria
SB1	Sunnerstabadet, Ekoln, Uppland	2016-08-24	++	+++	++		++															
SD1	Svandammen, Uppland	2016-09-26						+														Dominance of green algae
Så1	Svartån, Västerås, Västmanland	2016-09-27		+++		++																
Hå1	Utanför Lomma/Höje ån, Skåne	2016-08-28																				No cyanobacteria. Only *Eutreptiella sp.* an euglenophyte.
VB1	Vombsjön båtplatsen, Skåne	2016-08-31	++	+++	+	++																
VS1	Vombsjön, Västra sidan	2016-09-20		+++		++																High abundance of cryptophytes and bentic diatoms.
VSB1	Vårdsätrabadet, Ekoln, Mälaren	2016-08-28		+++																		
17-02	Stenholmen, Dalarö	2017-06-23																				Only pine pollen and diatoms
17-03	Hammarskogsbadet, Ekoln, Mälaren	2017-07-12	++																	+++		
17-04	Rännavägen, Gävle	2017-07-11																				Only marine dinoflagellate Heterocapsa found
17-05	Krönbadet, Vimmerby	2017-07-13	+++	++		++																

Sample name	Sampling site	Sampling Date	Dolichospermum	Microcystis	Aphanizomenon	Woronichinia	Planktolyngbya	Planktothrix	Cuspidothrix	Limnothrix	Gloeotrichia	Pseudanabaena	Snowella	Chroococcus	Nodularia	Radiocystis	Anabaenopsis	Merismopedia	Geitlerinema	Oscillatoria	Romeria	Comment
17-06	Ringsjön, Östra, Gamlabo	2017-07-13	++	+++	++									+								
17-07	Rörbacks Hamn	2017-07-12	+++					++														High abundance of akinetes
17-08	Herrö Pålsbo, Svegssjön, Sveg	2017-07-15	+++																			High abundance of akinetes
17-09	Strömsborgsvägen 72, Norrtälje	2017-07-20	+++		+++										++							
17-10	Hargsjön, Mjölby	2017-07-20	+++	++	++				++													
17-11	Svegsjön, Herrö	2017-07-20	+++																			High abundance of akinetes
17-12	Ruggen, Åkersberga	2017-07-23	+++			++																
17-13	Dämstasjön, Kramfors	2017-07-26	+++			++																
17-14	Bergsbadet, Linköping	2017-07-28									+++											
17-15	Sandvik, Linköping	2017-07-28	++	++	++						+++											more green algae and diatoms than cyanobacteria
17-16	Granön, Båtskärsnäs	2017-07-27	+++			++																High abundance of diatoms
17-17	Staka kraftverk, Flian	2017-07-28																				na
17-18	Gamla Lövöns kraftverk, Fångsjön	2017-07-26	+																			High abundance of green algae Botryococcus
17-19	Hålsjön, Alafors	2017-08-02	+	+++		+++								+								High abundance of green algae, diatoms and cryptophytes
17-20	Vimmesjöns badplats, Alafors	2017-08-02	++	++	+++																	
17-21	Sjön Anten, Alsingsås	2017-08-01	+++		++																	
17-22	Rånnavägs badplats, Surströmmasjön, Ulricehamn	2017-08-03	+++																			

**Sample name**	**Sampling site**	**Sampling Date**	Dolichospermum	Microcystis	Aphanizomenon	Woronichinia	Planktolyngbya	Planktothrix	Cuspidothrix	Limnothrix	Gloeotrichia	Pseudanabaena	Snowella	Chroococcus	Nodularia	Radiocystis	Anabaenopsis	Merismopedia	Geitlerinema	Oscillatoria	Romeria	**Comment**
17-23	Lofthammar, Västervik	2017-08-04									+++											Cells decomposed
17-24	Svanbergabadet, Norrtälje (Erken)	2017-08-08	+	+							+++											
17-25	Råvattenintaget till Rottne Vattenverk	2017-08-08																				No cyanobacteria. High abundance of diatoms
17-26	Öved, Vombsjön	2017-08-07																				No cyanobacteria. High abundance of diatoms.
17-27	Lammhults vattenverk, råvattenintaget, Stora Värmen	2017-08-08				+		+														High abundance of crypto-phytes and diatoms.
17-28	Ågesta, Stockholm, Magelungen	2017-08-08	++	++	+++	++		+	++				+	+								
17-29	Svinstadsjöns badplats, Linköping	2017-08-09	++	+++	++	++	+															
17-30	Glaningen sjö, Ludvika	2017-08-09																				Sample decoposed, not possible to identify cells.
17-31	Växjösjön, Växjö	2017-08-14	++		+++	++																No dominance of cyanobac-teria. More diatoms and cryptophytes. Also dinoflag-ellates, chrysophytes and green algae.
17-32	Södra Bergundasjön	2017-08-14		+++		++																
17-33	Övden, Alvesta kommun	2017-08-08	++																			Decomposed cells. Cyanobacteria with akinetes.
17-34	Trummen, Vaxsjö	2017-08-14	++	++		+++							+++			++						No dominance of cyanobac-teria. Diatoms more com-mon.

Sample name	Sampling site	Sampling Date	Dolichospermum	Microcystis	Aphanizomenon	Woronichinia	Planktolyngbya	Planktothrix	Cuspidothrix	Limnothrix	Gloeotrichia	Pseudanabaena	Snowella	Chroococcus	Nodularia	Radiocystis	Anabaenopsis	Merismopedia	Geitlerinema	Oscillatoria	Romeria	Comment
17-35	Tisarens utlopp, Hallsberg	2017-08-15	++	++	+++	++		+														
17-36	Virens utlopp, Katrineholm	2017-08-15	++	++	+++	++	+	++		+												
17-37	Ellestadssjön, Sjöbo	2017-08-16	+	+	+++				+								+					
17-38	Dånviken, Rönninge	2017-08-17	++	++	++	++	+							+								
17-39	Yngaren, Nyköping	2017-08-17	++	++	+++	++		+			++			+								
17-40	Runnviken	2017-08-17	++	++	+++	++		++	++													
17-41	Stehags badplats, Västra Ringsjön	2017-08-18	++	++		++	+	+++	++				+									
17-42	Roxen, Linköping	2017-08-18									+++											
17-43	Regnaren, Nyköping	2017-08-22	+++	++	++	++																
17-44	Börringesjön, Svedala	2017-08-22			++		+++	++	++													
17-45	Finjasjön, Skåne	2017-08-22		+	+++	+		+														
17-46	Hallbosjön, Nyköping	2017-08-22	++	++	++	++	+			+	+++											Gloeotrichia only filaments, not whole colonies
17-47	Fjällfotasjön	2017-08-22	+	+	++		+++			+						+						
17-48	Arkelstorpsviken, Skåne	2017-08-22	+++	++			+						+									
17-49	Hornsundssjön, Flen	2017-08-22	+++	++	++	++	+	++		+												
17-50	Hjälmaren	2017-08-22			++		++			+++									++			
17-51	Vombsjön	2017-08-23	++	+++	++	++																
17-52	Väsbyviken, Torshälla	2017-08-22	++	++	+++	++	+	+	++	+		+					++					High abundance of dinoflag-ellates and diatoms
17-53	Västeråsfjärden, Mälaren	2017-08-23	++	+++	++	++								+								
17-54	Havgårdssjön, Dävelstorp	2017-08-23	+++	++	++																	

Sample name	Sampling site	Sampling Date	Dolichospermum	Microcystis	Aphanizomenon	Woronichinia	Planktolyngbya	Planktothrix	Cuspidothrix	Limnothrix	Gloeotrichia	Pseudanabaena	Snowella	Chroococcus	Nodularia	Radiocystis	Anabaenopsis	Merismopedia	Geitlerinema	Oscillatoria	Romeria	Comment
17-55	Krageholmssjön rastplats (Hästhagsv.)	2017-08-23	+++	++	++																	
17-56	Näsbyholmssjön, Skåne	2017-08-23	+		+++																	High abundance of akinetes
17-57	Östra Ringsjön, Skåne	2017-08-23	++	++	++	++	+	++	++				+									High abundance of diatoms and green algae
17-58	Råbelövssjön, Ekestad	2017-08-22	+++	+++	++																	Cells decomposed
17-59	Vadsbrosjön, Nyköping	2017-08-23	+++	++	+++	++			++													
17-60	Fält Danviken, Salems kommun	2017-08-24	+++	++	++	++																
17-62	Danviken, Salems kommun	2017-09-02	+++	++	++	++																
17-63	Storsjön, Gåsnäs, Kramfors	2017-09-04	+++													+						
17-64	Hjorten, Västervik	2017-08-28																				na
17-65	Hjorten, Västervik	2017-08-31	++	++							+++											
17-66	Vombsjön, Östra Vombs badplats, Skåne	2017-09-06		+++	+																	
17-67	Finjasjön, Björkviken, Tormstorp	2017-09-06			+++																	
17-68	Östra Ringsjön, Skåne	2017-09-06	++		+++	++		++														
17-69	Ringsjö	2017-09-11	++	++	+++	++		++														No cyanobacterial dominance. Leaves of *Lemna* in the bottle
17-70	Långhalsen	2017-09-11	++	++	+++	++	+		++	+		+										
17-71	Dånviken, Rönningen	2017-09-08	+++	++	++	++																
17-72	Västerås gästhamn, Mälaren	2017-09-13																				na
17-73	Linnéasjön, Nybro	2017-09-18	+++																			Colonies attach to larger plant material

**Sample name**	**Sampling site**	**Sampling Date**	Dolichospermum	Microcystis	Aphanizomenon	Woronichinia	Planktolyngbya	Planktothrix	Cuspidothrix	Limnothrix	Gloeotrichia	Pseudanabaena	Snowella	Chroococcus	Nodularia	Radiocystis	Anabaenopsis	Merismopedia	Geitlerinema	Oscillatoria	Romeria	**Comment**
17-74	Sövdesjön, Sjöbo	2017-09-18	+++	++	++	++		++	++													
17-75	Fiskekroken, Härnösand	2017-09-28			+++										+++							Cells decomposed
17-76	Sträsundsviken, Köpmannaholmen	2017-10-02													+++							Cells decomposed. High abundance of diatoms.
